# Supplementary material for: Meiotic cohesion requires Sirt1 and preserving its activity in aging oocytes reduces missegregation
Source: EMBO Rep. 2025 Nov 10;26(24):6121–40. doi: 10.1038/s44319-025-00634-y (PMC12714828; doi:10.1038/s44319-025-00634-y)
Supplement: Supplementary file 1 — Appendix [file 44319_2025_634_MOESM1_ESM.pdf]

APPENDIX

Appendix Table S1. Age-dependent X-chromosome NDJ in oocytes is suppressed by feeding mothers the Sirt1 activator, SRT1720 **p2**

Appendix Table S2. Fly Stocks and Genotypes **p3**

References for Appendix **p4**

Appendix Table S1: Age-dependent X-chromosome NDJ in oocytes is suppressed by feeding mothers the Sirt1 activator, SRT1720

Condition	% NDJ	% Diplo *(#)*	% Nullo *(#)*	N *(fertility)*	P-value
Replicate 1					
Aged, DMSO	15.26	7.07 *(57)*	8.19 *(66)*	1489 *(37.2)*	7.78×10^{-6}
Non-Aged, DMSO	7.83	4.18 *(31)*	3.64 *(27)*	1424 *(39.6)*	
Aged, SRT1720	12.79	6.01 *(46)*	6.79 *(52)*	1434 *(35.9)*	0.126
Non-Aged, SRT 1720	10.19	4.13 *(30)*	6.06 *(44)*	1378 *(34.5)*	
Replicate 2					
Aged, DMSO	15.12	8.21 *(38)*	6.91 *(32)*	856 *(23.8)*	5.35×10^{-4}
Non-Aged, DMSO	8.02	4.16 *(27)*	3.86 *(25)*	1245 *(31.1)*	
Aged, SRT1720	11.43	5.87 *(37)*	5.56 *(35)*	1188 *(27.0)*	0.109
Non-Aged, SRT 1720	8.70	5.36 *(37)*	3.34 *(23)*	1320 *(33.0)*	
Replicate 3					
Aged, DMSO	15.42	8.57 *(35)*	6.85 *(28)*	754 *(18.9)*	2.48×10^{-3}
Non-Aged, DMSO	8.51	3.40 *(16)*	5.11 *(24)*	900 *(22.5)*	
Aged, SRT1720	9.56	3.74 *(18)*	5.82 *(28)*	916 *(22.9)*	0.232
Non-Aged, SRT 1720	7.43	2.18 *(12)*	5.25 *(29)*	1063 *(26.6)*	

In all three experiments, the genotype of mothers was: *y w/y w; + : $mtrm^{KG}$ smc1Δ / +.*
# indicates number of progeny recovered for each category.
N equals the total number of progeny scored.
Fertility equals the number of progeny per female.
P-values were calculated for pairs using the calculator developed by Gilliland and colleagues (Zeng *et al*, 2010).

Appendix Table S2. Fly Stocks and Genotypes

Genotype	Hairpin	Abbreviation	Source	Bickel Stock #
y^{1} *sc*^{1} *v*^{1} *; P{y*$^{+t7.7}$ *v*$^{+t1.8}$*= TRiP.* HMJ21708 =***Sirt1***^{V20}*}attP40; +*	SH022-B06 Valium 20	*Sirt1*$^{SH022-B06}$	BL #36614	H-087 H-213[#]
y ; P{y$^{+t7.7}$ *v*$^{+t1.8}$*= TRiP.* HMJ21708 =***Sirt1***^{V20}*}attP40; +*	SH022-B06 Valium 20	*Sirt1*$^{SH022-B06}$	Bickel lab derivative of H-087	I-554
y^{1} *sc* v*^{1} *; + ; P{y*$^{+t7.7}$ *v*$^{+t1.8}$*=TRiP HMS00484=****Sirt1***^{V20}*}attP2*	SH00806 Valium 20	*Sirt1*^{SH00806}	BL #32481	H-084 H-214[#]
y ; + ; P{y$^{+t7.7}$ *v*$^{+t1.8}$*=TRiP HMS00484=****Sirt1***^{V20}*}attP2*	SH00806 Valium 20	*Sirt1*^{SH00806}	Bickel lab derivative of H-084	I-543
y sc cv v f car*/FM7a/B*^{S}*Y ; + ;* ***mtrm***^{KG0805} *P{w*$^{+mC}$*=****matα4****-GAL4-VP16}V37/TM3,Sb(Ser)*		*y sc cv v f car; mtrm*^{KG} *matα*	Bickel Lab (Perkins *et al*, 2016)	M-834
y sc cv v f car*/FM7a/B*^{S}*Y ; + ;* ***mtrm***^{KG0805}*/TM3,Sb(Ser)*		*y sc cv v f car; mtrm*^{KG}	Bickel Lab (Perkins *et al.*, 2016)	M-835
C(1)RM, y^{2}*, su(w*^{a}*) w*^{a} */* ***X^Y,v f B***		*X^Y, Bar*	BL #700	C-200
y^{1} *Df(1)w*^{67c23} *; + ; +*		*y w*		A-062
w; + ; P{w*$^{+mC}$*=****matα4****-GAL4-VP16}V37*		*matα*	BL #7063	T-273
+ ; ***sirt1***$^{5.26}$ *cn*^{1} *; +*		*sirt1*$^{5.26}$	BL #32657 (Newman *et al*, 2002)	B-193
+ ; ***sirt1***$^{4.5}$ *cn*^{1}*/SM6b, P{ry*$^{+t7.2}$*=eve-lacZ8.0}SB1; +*		*sirt1*$^{4.5}$	BL #32568 (Newman *et al.*, 2002)	B-194
y w ; + ; FRT82B cu sr ***smc1Δ***^{ex46}*/ TM3 Sb*			Hawley Lab	M-744
y^{1}*; + ; P{y*$^{+mDint2}$ *w*$^{BR.E.BR}$*=SUPorP} Exo70*^{KG08051} ***mtrm***^{KG0805} *ry*^{506} */ TM3,Sb*^{1}*Ser*^{1}			BL #14932	M-755
y w / B[S]Y; + ; ***mtrm***^{KG0805} *sr* ***smc1Δ***^{ex46} */ TM3, Sb, Ser*		*mtrm*^{KG} *smc1Δ*	Bickel lab derivative of M-744 and M-755	M-822

BL = Bloomington Drosophila Stock Center
[#]Over the course of this study, stock was reordered from Bloomington and given a new Bickel Stock #

REFERENCES for APPENDIX

Newman BL, Lundblad JR, Chen Y, Smolik SM (2002) A Drosophila homologue of Sir2 modifies position-effect variegation but does not affect life span. *Genetics* 162: 1675-1685

Perkins AT, Das TM, Panzera LC, Bickel SE (2016) Oxidative stress in oocytes during midprophase induces premature loss of cohesion and chromosome segregation errors. *Proc Natl Acad Sci U S A* 113: E6823-E6830

Zeng Y, Li H, Schweppe NM, Hawley RS, Gilliland WD (2010) Statistical analysis of nondisjunction assays in Drosophila. *Genetics* 186: 505-513
