# Supplementary material for: Meiotic cohesion requires Sirt1 and preserving its activity in aging oocytes reduces missegregation
Source: EMBO Rep. 2025 Nov 10;26(24):6121–40. doi: 10.1038/s44319-025-00634-y (PMC12714828; doi:10.1038/s44319-025-00634-y)
Supplement: Supplementary file 4 — Source data Fig. 2 [file 44319_2025_634_MOESM4_ESM.zip › Figure 2/Fig 2B/0. Read Me.rtf]

All images are maximum intensity projections of confocal Z series
