# Supplementary figures and images for: Meiotic cohesion requires Sirt1 and preserving its activity in aging oocytes reduces missegregation
Source: EMBO Rep. 2025 Nov 10;26(24):6121–40. doi: 10.1038/s44319-025-00634-y (PMC12714828; doi:10.1038/s44319-025-00634-y)

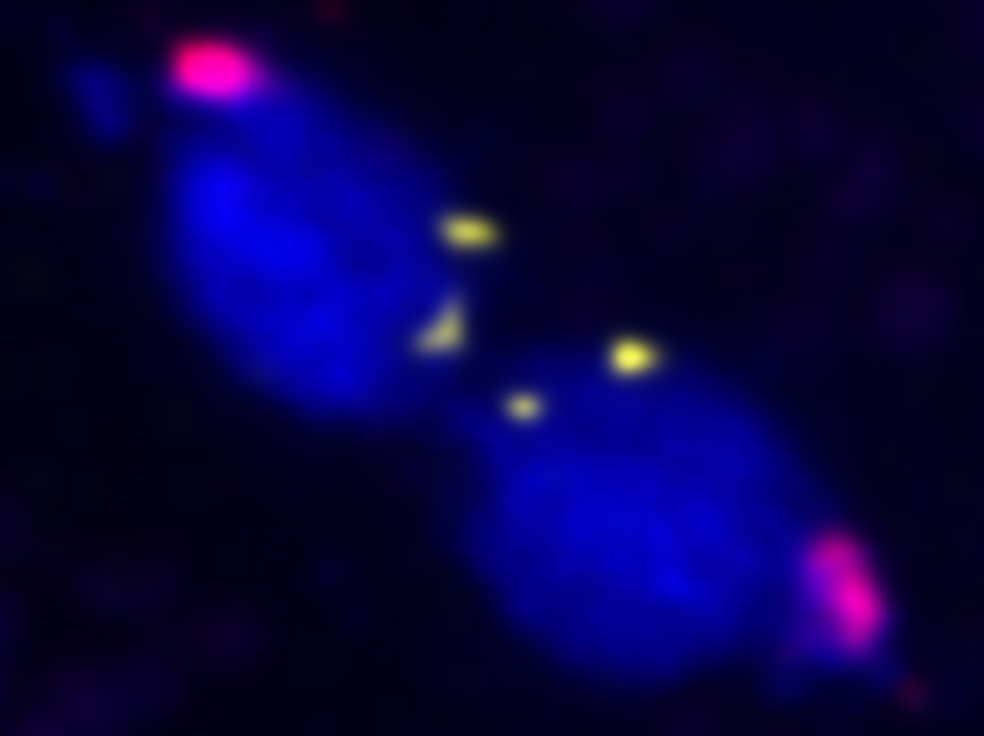

Supplement: Supplementary file 4 — Source data Fig. 2 [file 44319_2025_634_MOESM4_ESM.zip › Figure 2/Fig 2B/4 arm spots ZM-077-021.nd2.tif]

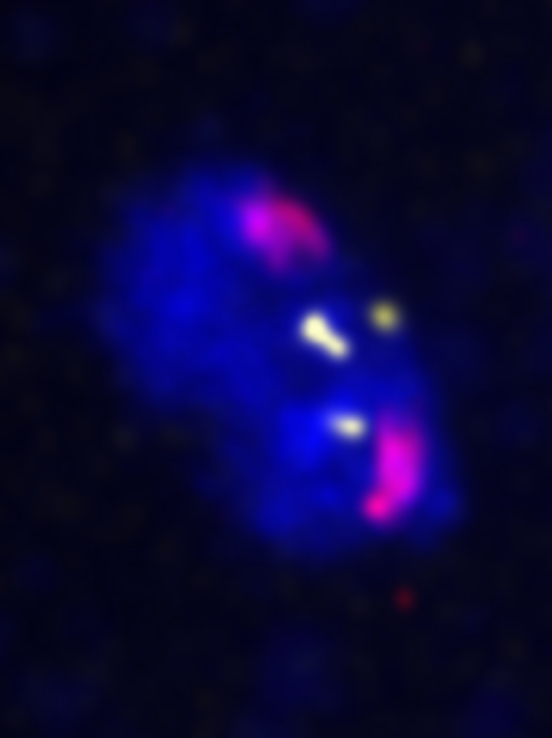

Supplement: Supplementary file 4 — Source data Fig. 2 [file 44319_2025_634_MOESM4_ESM.zip › Figure 2/Fig 2B/3 arm spots ZM-075-004.nd2.tif]

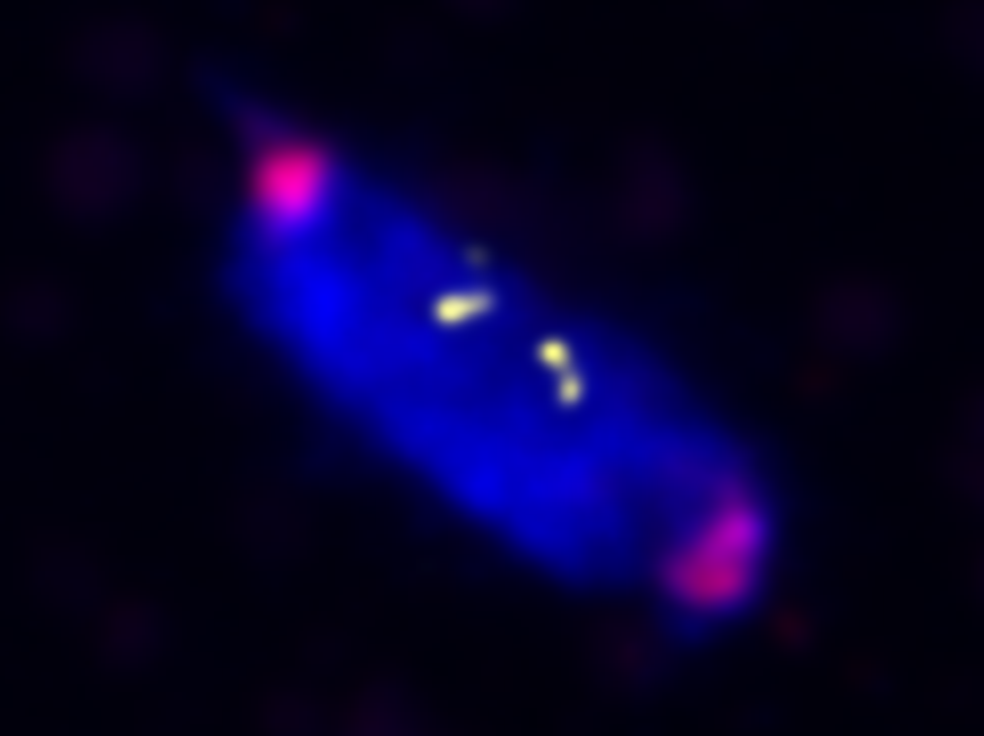

Supplement: Supplementary file 4 — Source data Fig. 2 [file 44319_2025_634_MOESM4_ESM.zip › Figure 2/Fig 2B/2 arm spots ZM-077-020.nd2.tif]

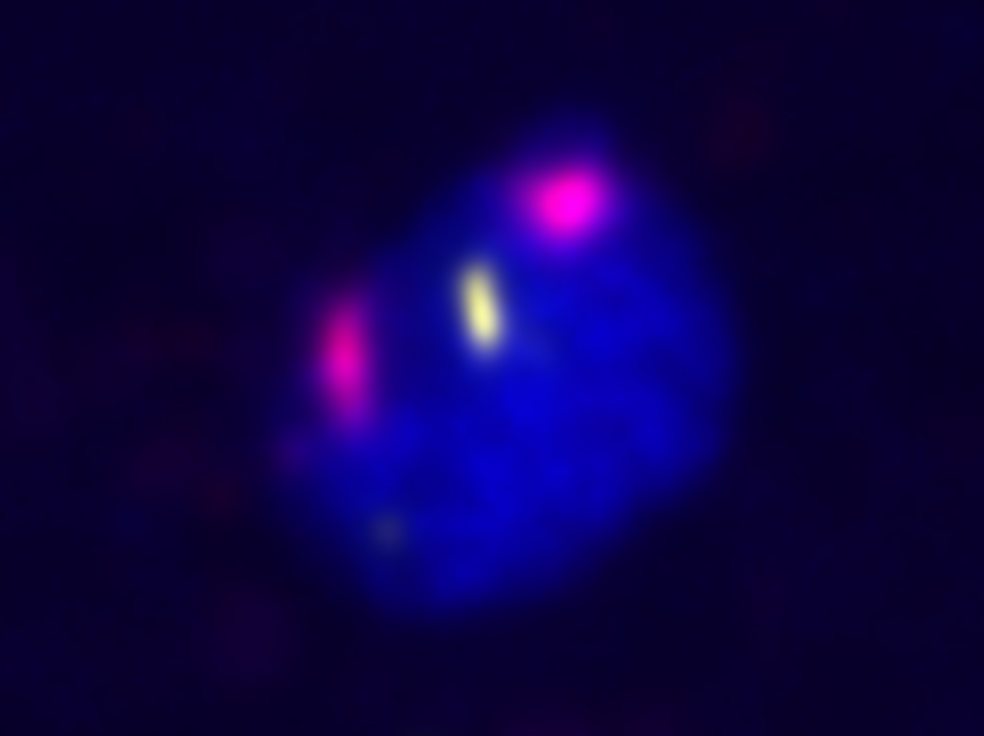

Supplement: Supplementary file 4 — Source data Fig. 2 [file 44319_2025_634_MOESM4_ESM.zip › Figure 2/Fig 2B/1 arm spot ZM-077-035.nd2.tif]

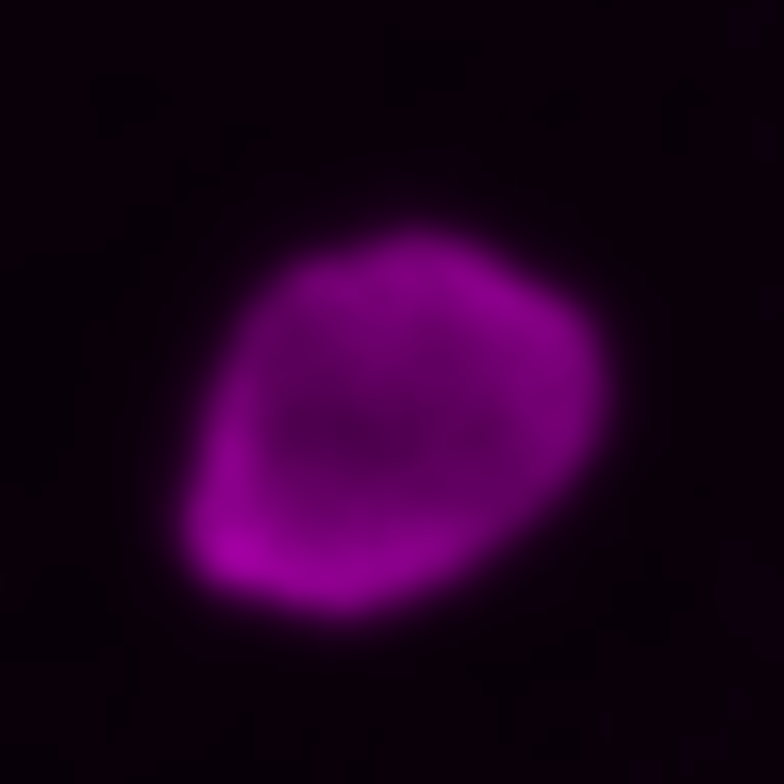

Supplement: Supplementary file 5 — Source data Fig. 3 [file 44319_2025_634_MOESM5_ESM.zip › Figure 3/Fig 3A/Sirt1 KD/H4K16ac-SIRT1-KD-004.nd2.tif]

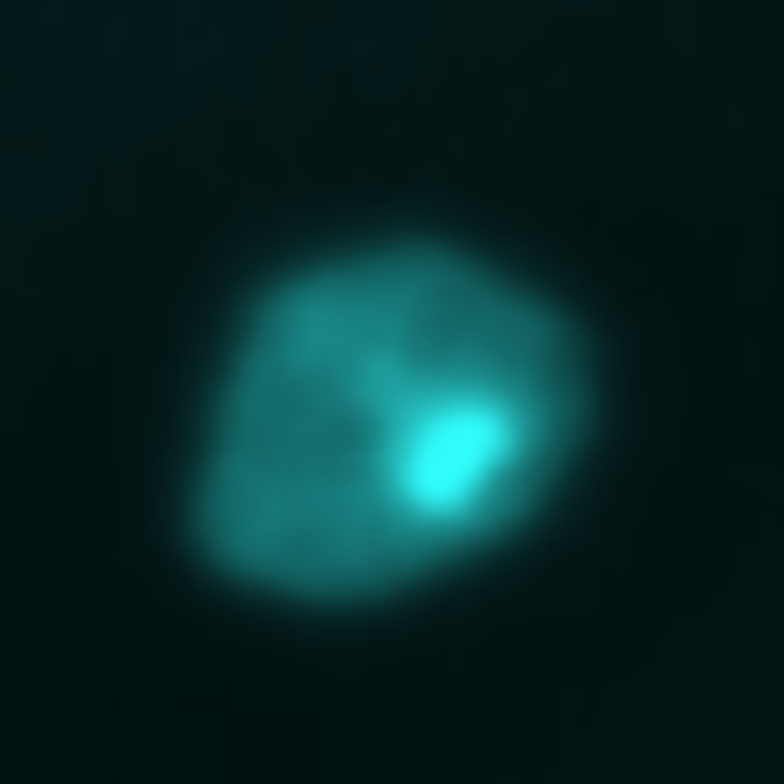

Supplement: Supplementary file 5 — Source data Fig. 3 [file 44319_2025_634_MOESM5_ESM.zip › Figure 3/Fig 3A/Sirt1 KD/DNA-SIRT1-KD-004.nd2.tif]

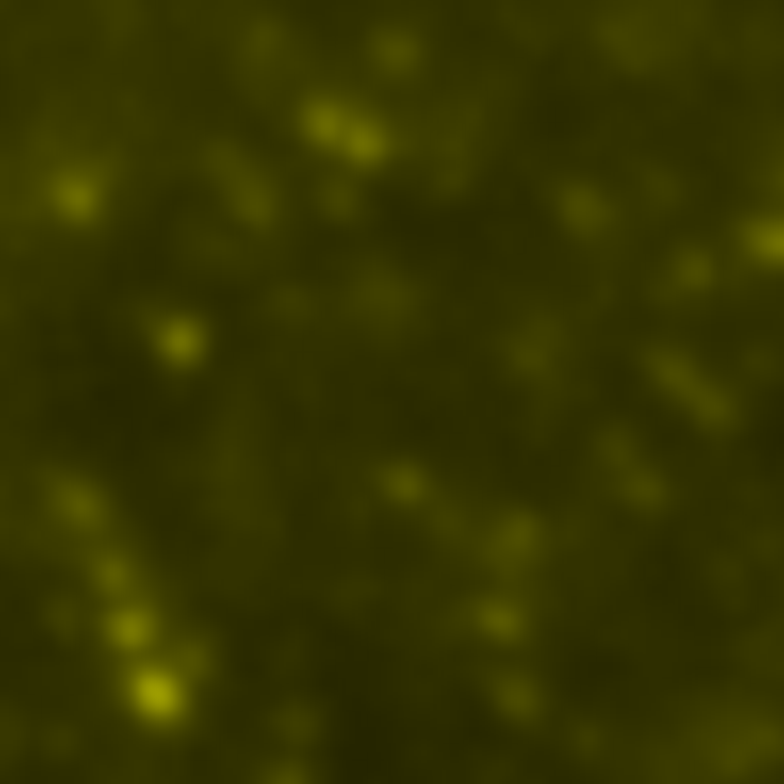

Supplement: Supplementary file 5 — Source data Fig. 3 [file 44319_2025_634_MOESM5_ESM.zip › Figure 3/Fig 3A/Sirt1 KD/Sirt1-SIRT1-KD-004.nd2.tif]

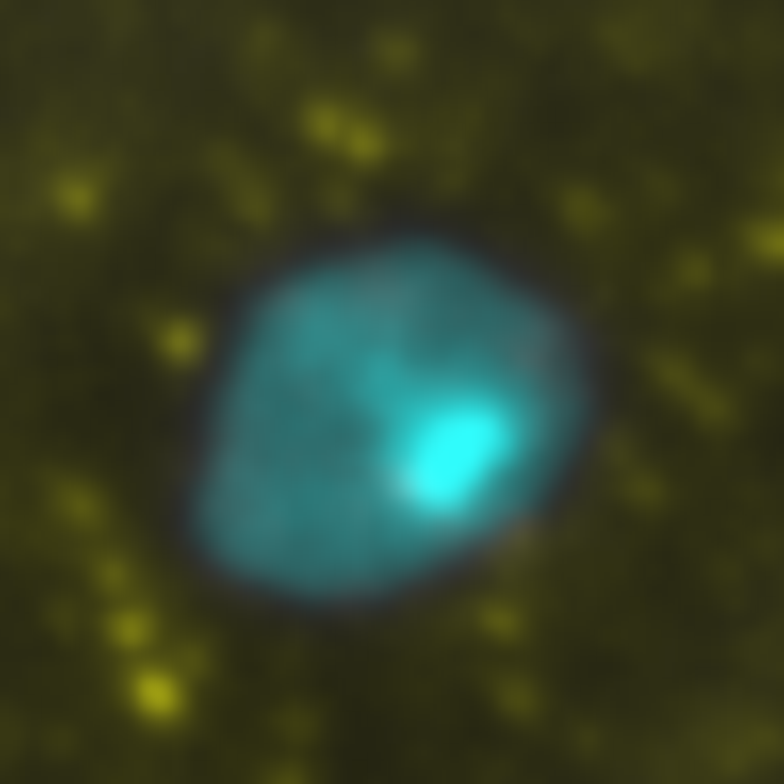

Supplement: Supplementary file 5 — Source data Fig. 3 [file 44319_2025_634_MOESM5_ESM.zip › Figure 3/Fig 3A/Sirt1 KD/S+DNA-SIRT1-KD-004.nd2.tif]

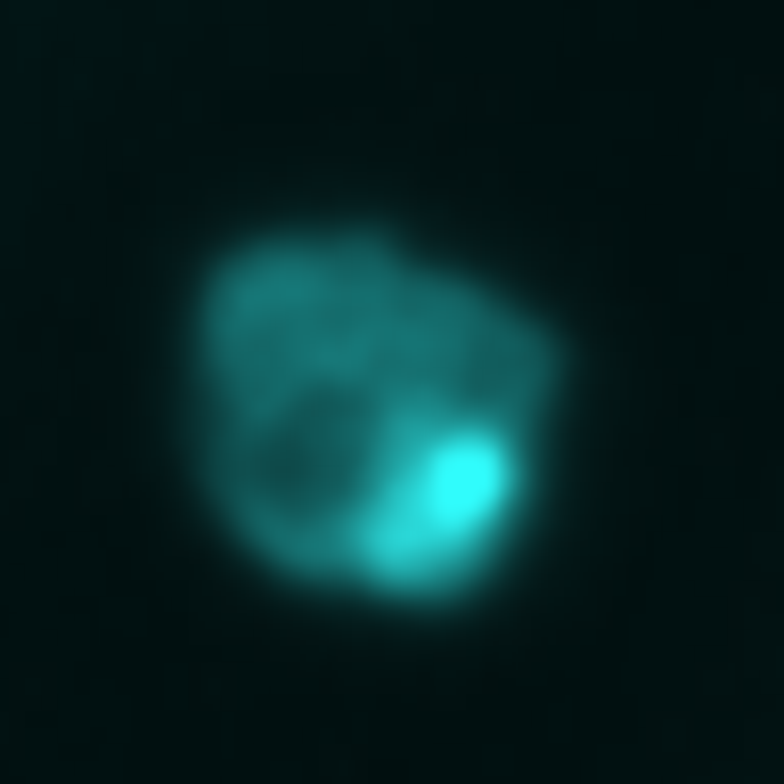

Supplement: Supplementary file 5 — Source data Fig. 3 [file 44319_2025_634_MOESM5_ESM.zip › Figure 3/Fig 3A/Sirt1 null/DNA-SIRT1-null-002.nd2.tif]

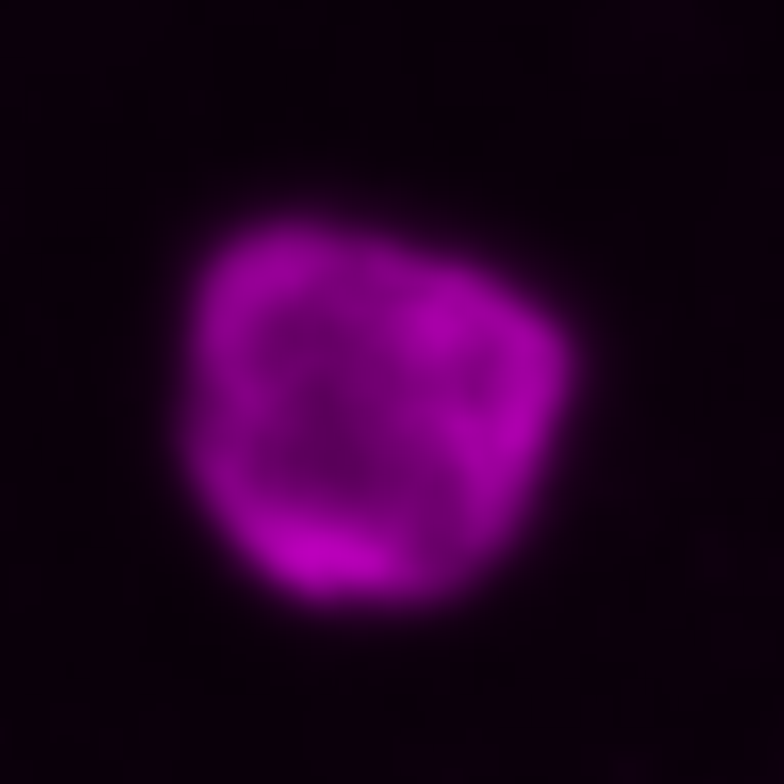

Supplement: Supplementary file 5 — Source data Fig. 3 [file 44319_2025_634_MOESM5_ESM.zip › Figure 3/Fig 3A/Sirt1 null/H4K16ac-SIRT1-null-002.nd2.tif]

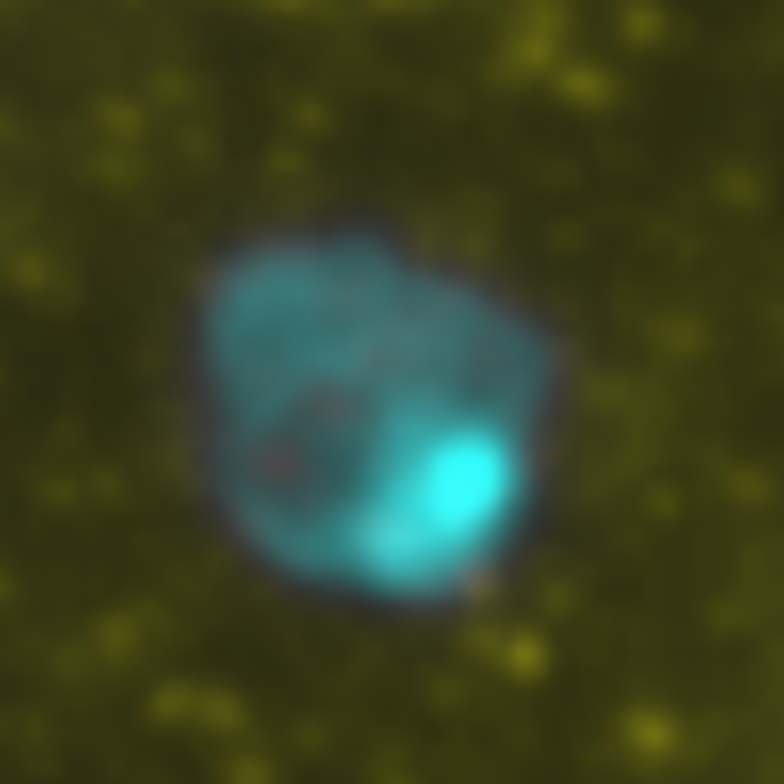

Supplement: Supplementary file 5 — Source data Fig. 3 [file 44319_2025_634_MOESM5_ESM.zip › Figure 3/Fig 3A/Sirt1 null/S+DNA-SIRT1-null-002.nd2.tif]

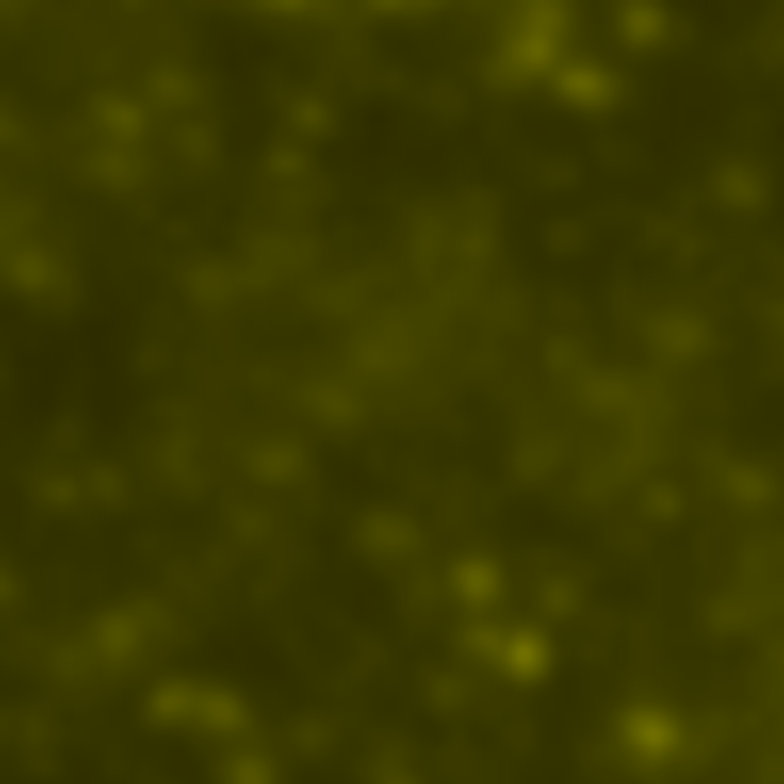

Supplement: Supplementary file 5 — Source data Fig. 3 [file 44319_2025_634_MOESM5_ESM.zip › Figure 3/Fig 3A/Sirt1 null/Sirt1-SIRT1-null-002.nd2.tif]

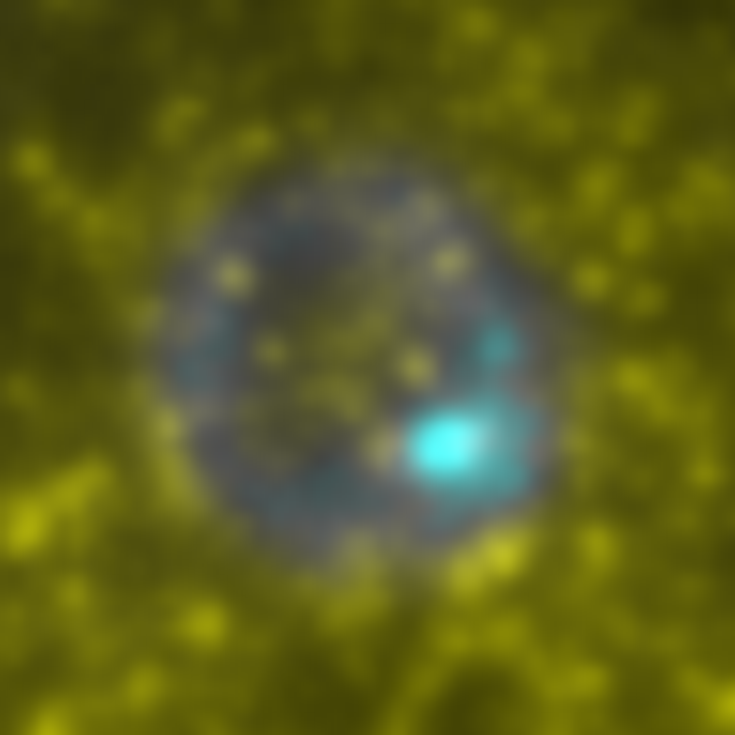

Supplement: Supplementary file 5 — Source data Fig. 3 [file 44319_2025_634_MOESM5_ESM.zip › Figure 3/Fig 3A/Sirt1 Control/S+DNA-SIRT1-ctrl-015.nd2.tif]

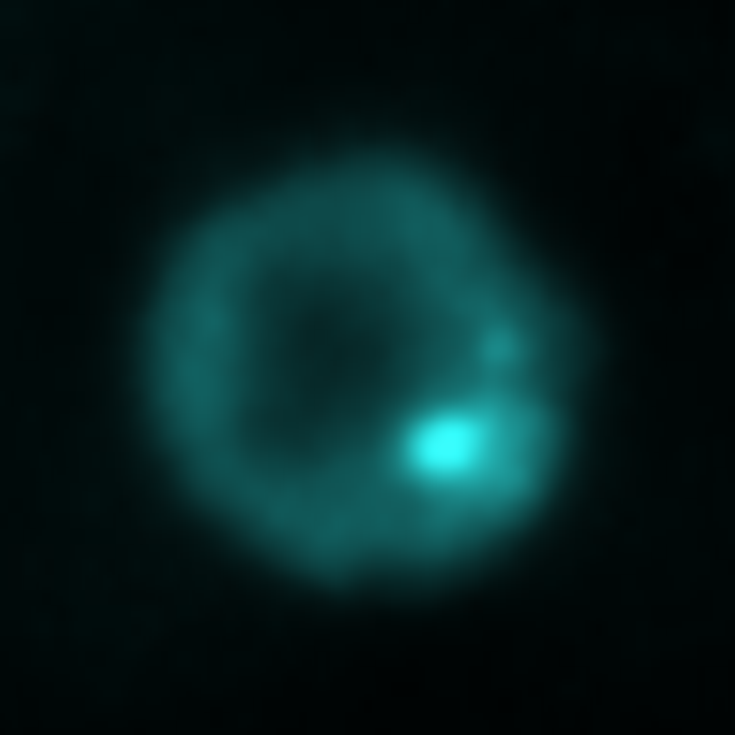

Supplement: Supplementary file 5 — Source data Fig. 3 [file 44319_2025_634_MOESM5_ESM.zip › Figure 3/Fig 3A/Sirt1 Control/DNA-SIRT1-ctrl-015.nd2.tif]

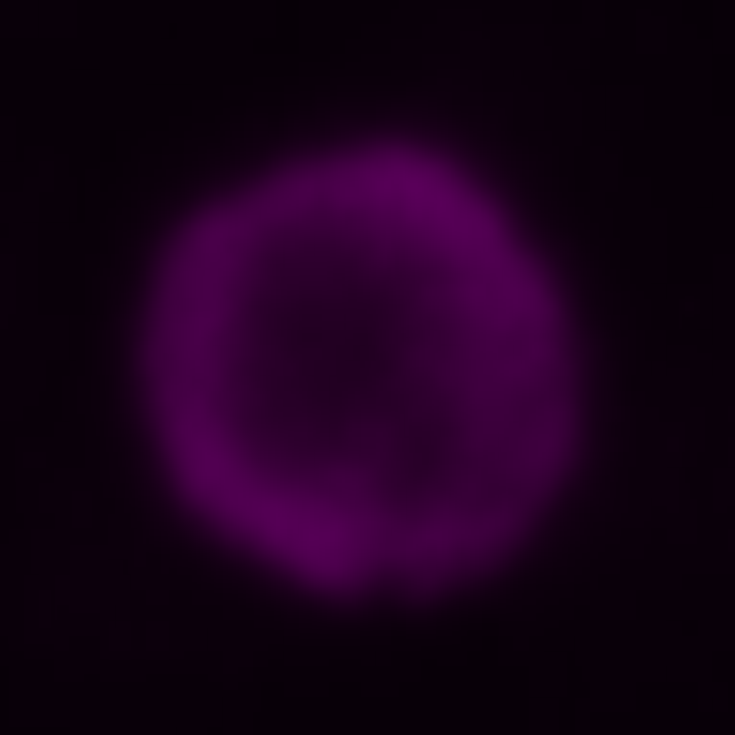

Supplement: Supplementary file 5 — Source data Fig. 3 [file 44319_2025_634_MOESM5_ESM.zip › Figure 3/Fig 3A/Sirt1 Control/H4K16ac-SIRT1-ctrl-015.nd2.tif]

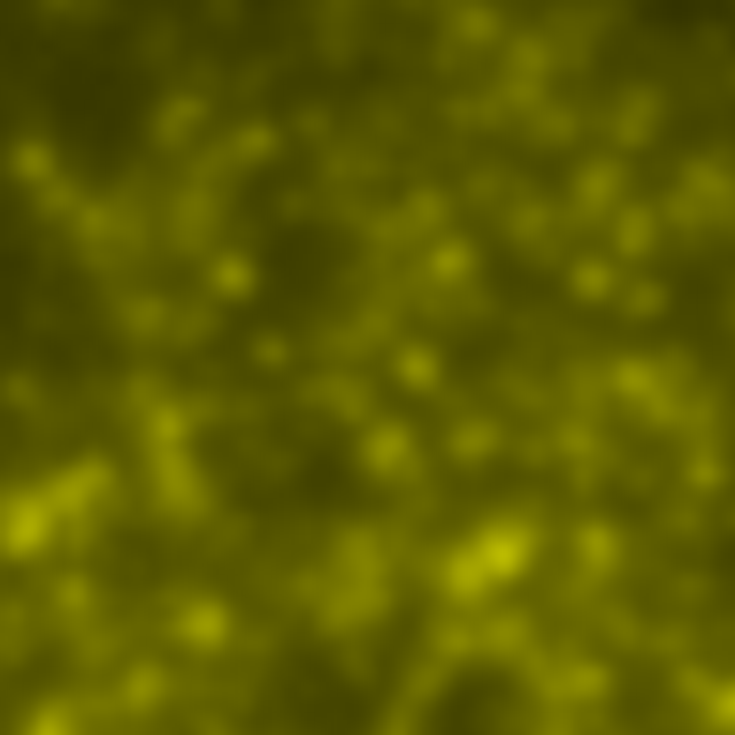

Supplement: Supplementary file 5 — Source data Fig. 3 [file 44319_2025_634_MOESM5_ESM.zip › Figure 3/Fig 3A/Sirt1 Control/Sirt1-SIRT1-ctrl-015.nd2.tif]

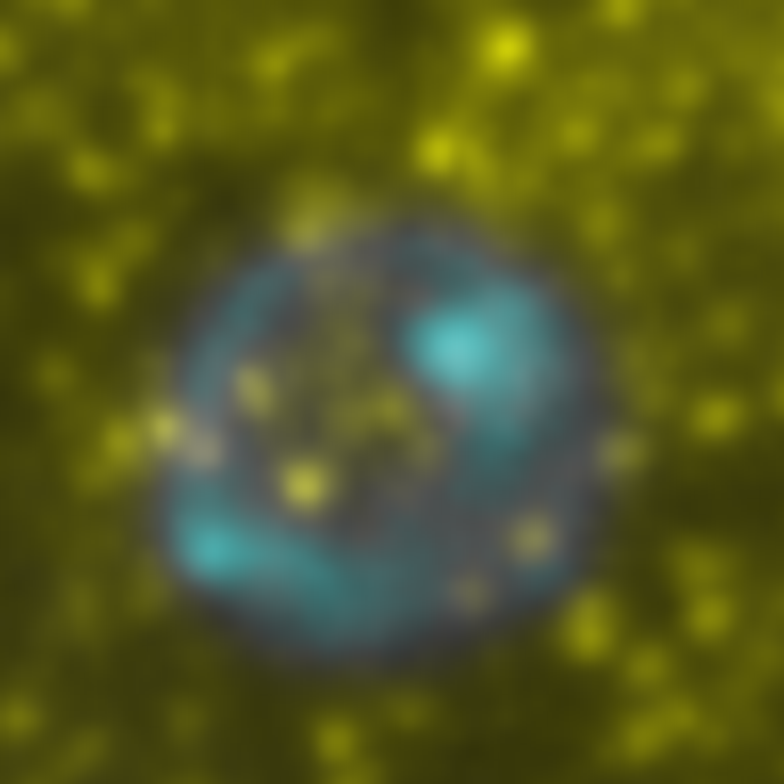

Supplement: Supplementary file 6 — Source data Fig. 4 [file 44319_2025_634_MOESM6_ESM.zip › Figure 4/Fig 4A Anti-Sirt1/Non-aged/Merge-Non-aged-013.nd2.tif]

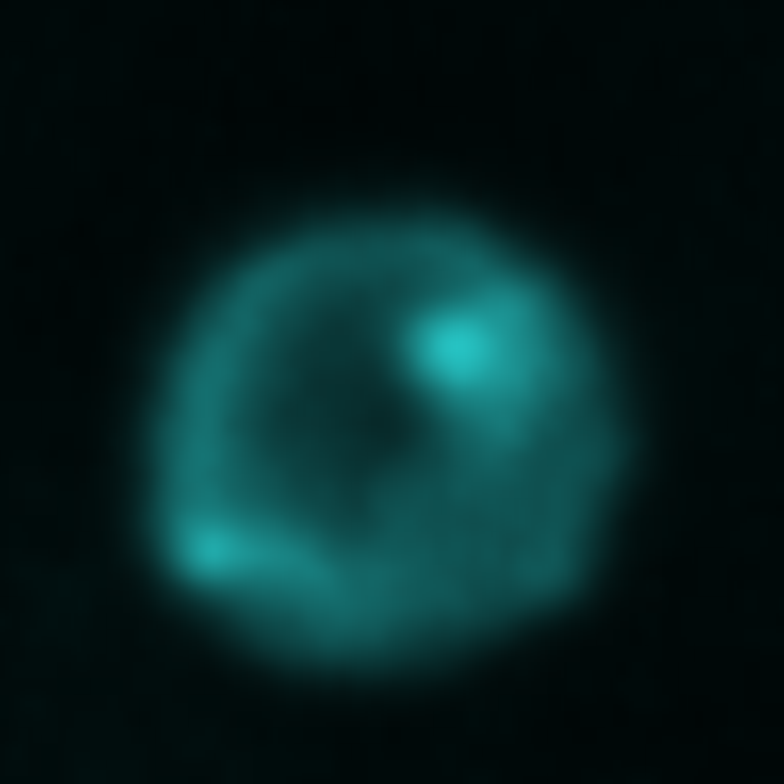

Supplement: Supplementary file 6 — Source data Fig. 4 [file 44319_2025_634_MOESM6_ESM.zip › Figure 4/Fig 4A Anti-Sirt1/Non-aged/DNA-Non-aged-013.nd2.tif]

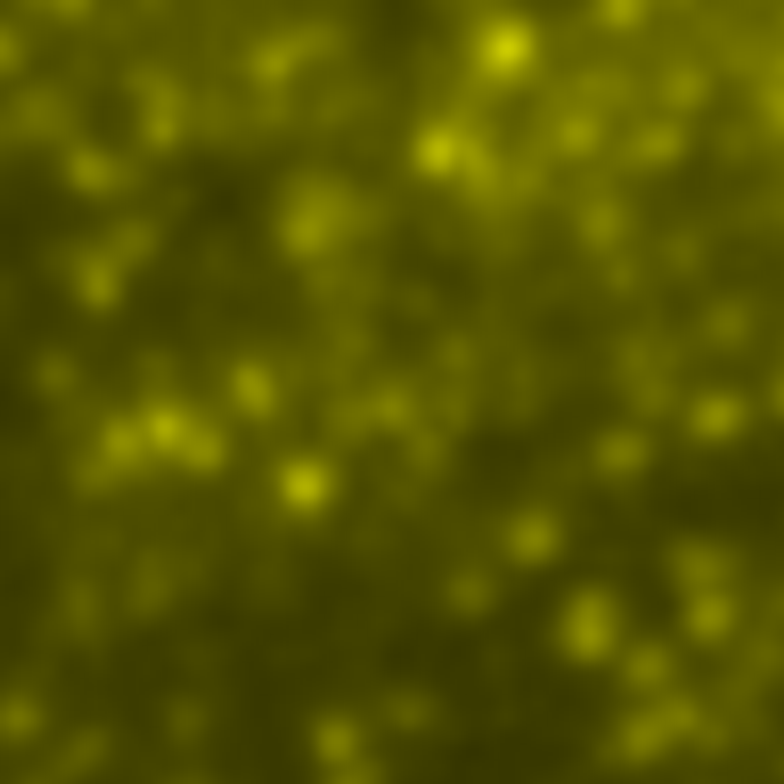

Supplement: Supplementary file 6 — Source data Fig. 4 [file 44319_2025_634_MOESM6_ESM.zip › Figure 4/Fig 4A Anti-Sirt1/Non-aged/Sirt1-Non-aged-013.nd2.tif]

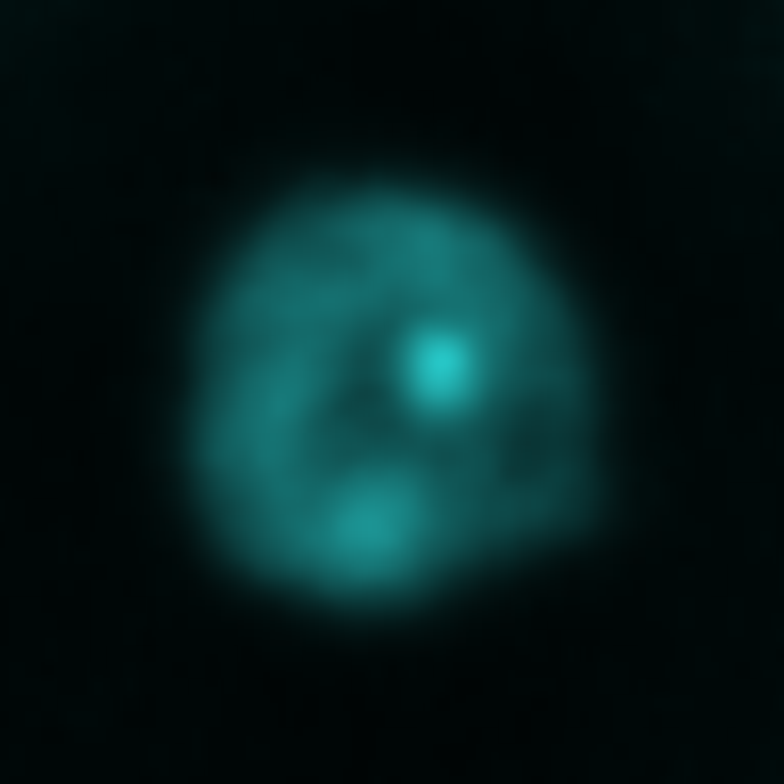

Supplement: Supplementary file 6 — Source data Fig. 4 [file 44319_2025_634_MOESM6_ESM.zip › Figure 4/Fig 4A Anti-Sirt1/Null/DNA-SIRT1 null-015.nd2.tif]

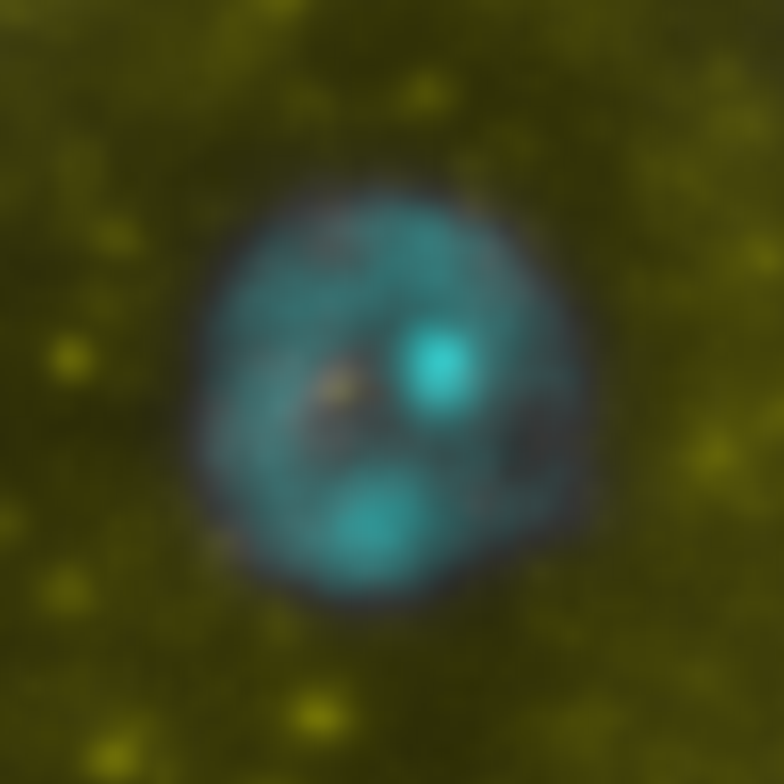

Supplement: Supplementary file 6 — Source data Fig. 4 [file 44319_2025_634_MOESM6_ESM.zip › Figure 4/Fig 4A Anti-Sirt1/Null/Merge-SIRT1 null-015.nd2.tif]

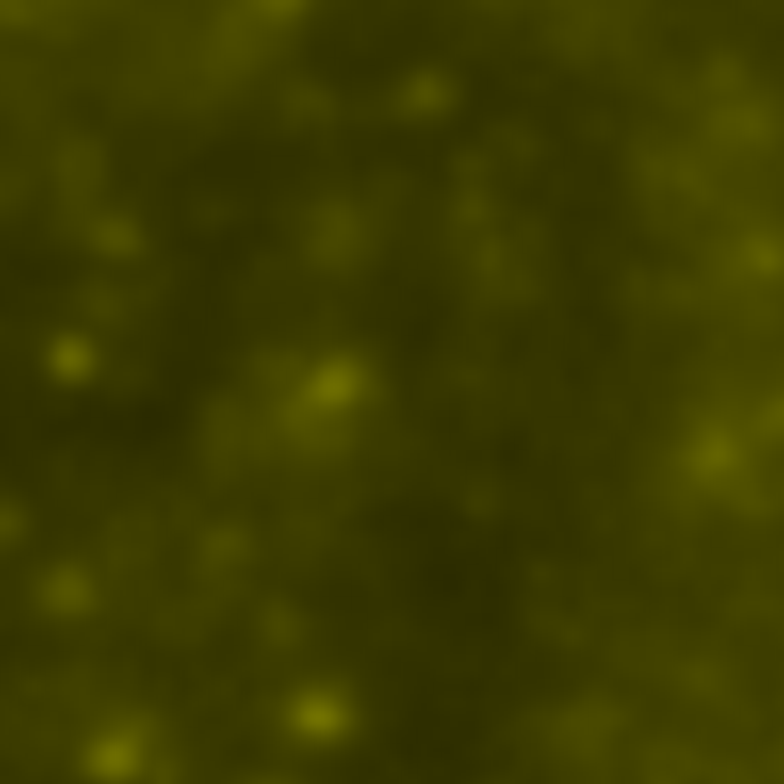

Supplement: Supplementary file 6 — Source data Fig. 4 [file 44319_2025_634_MOESM6_ESM.zip › Figure 4/Fig 4A Anti-Sirt1/Null/Sirt1-SIRT1 null-015.nd2.tif]

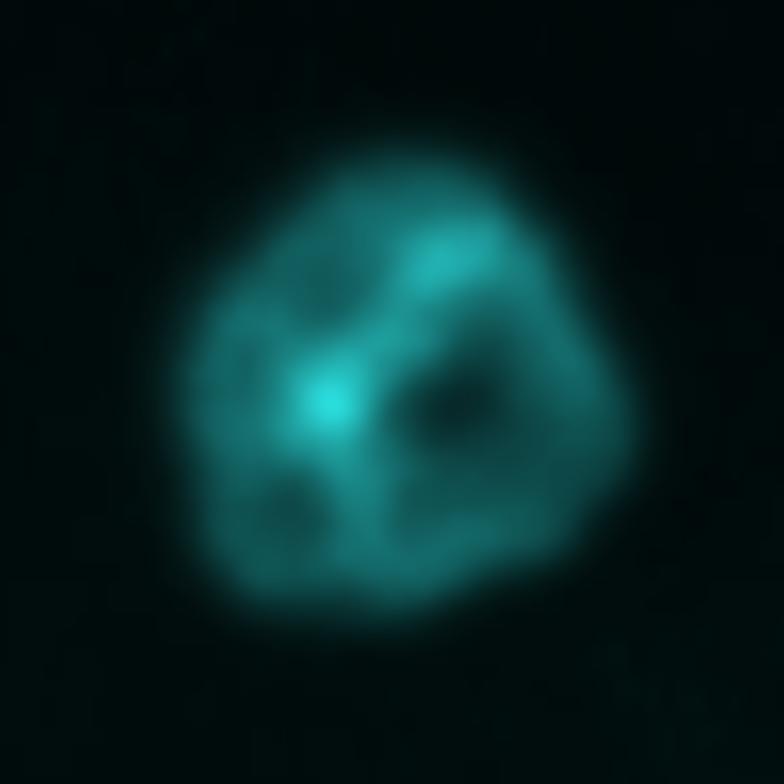

Supplement: Supplementary file 6 — Source data Fig. 4 [file 44319_2025_634_MOESM6_ESM.zip › Figure 4/Fig 4A Anti-Sirt1/Aged/DNA-Aged-002.nd2.tif]

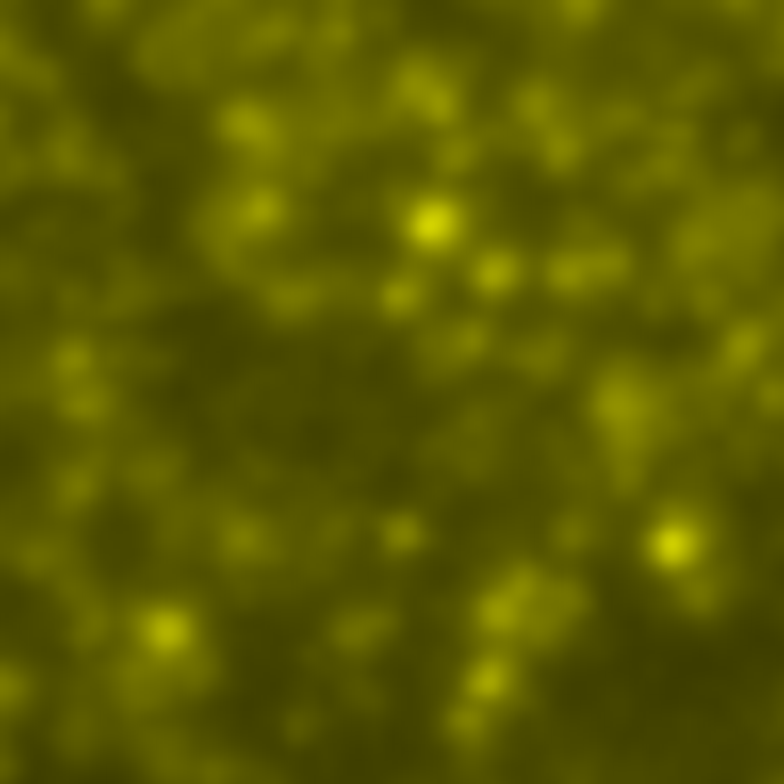

Supplement: Supplementary file 6 — Source data Fig. 4 [file 44319_2025_634_MOESM6_ESM.zip › Figure 4/Fig 4A Anti-Sirt1/Aged/Sirt1-Aged-002.nd2.tif]

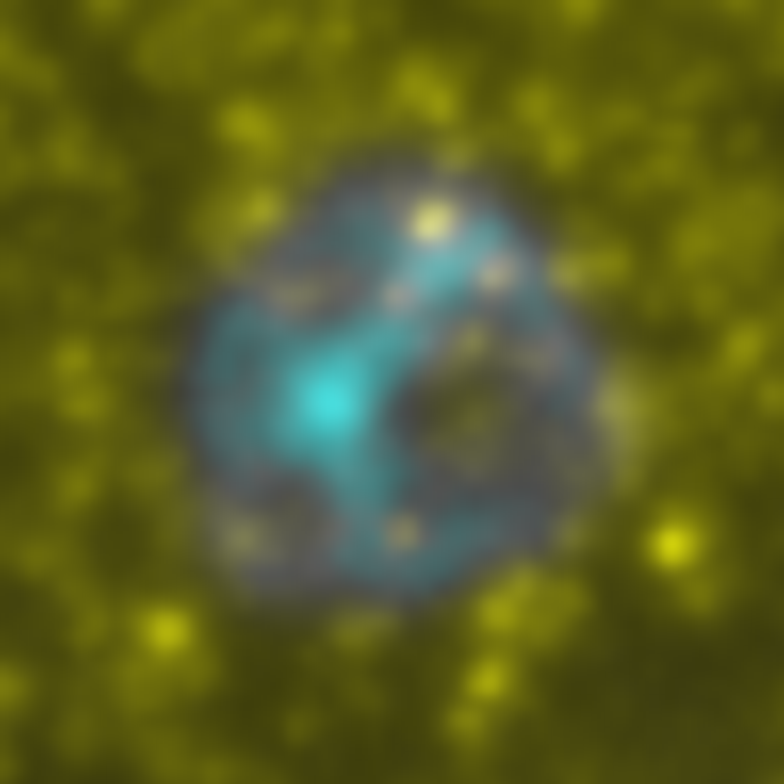

Supplement: Supplementary file 6 — Source data Fig. 4 [file 44319_2025_634_MOESM6_ESM.zip › Figure 4/Fig 4A Anti-Sirt1/Aged/Merge-Aged-002.nd2.tif]

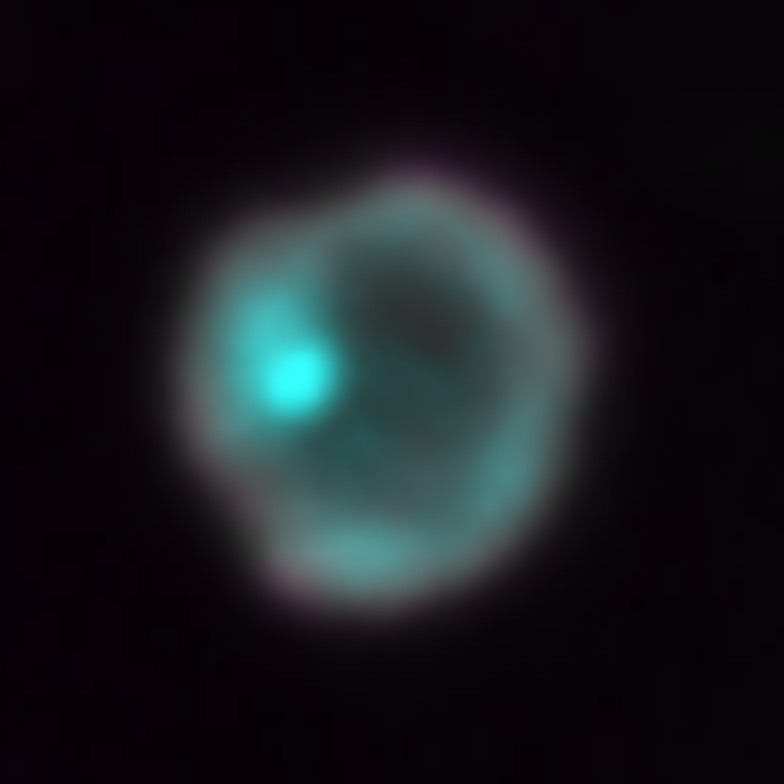

Supplement: Supplementary file 6 — Source data Fig. 4 [file 44319_2025_634_MOESM6_ESM.zip › Figure 4/Fig 4C Anti-H4K16ac/Non-Aged/Merge-Non-aged-010.nd2.tif]

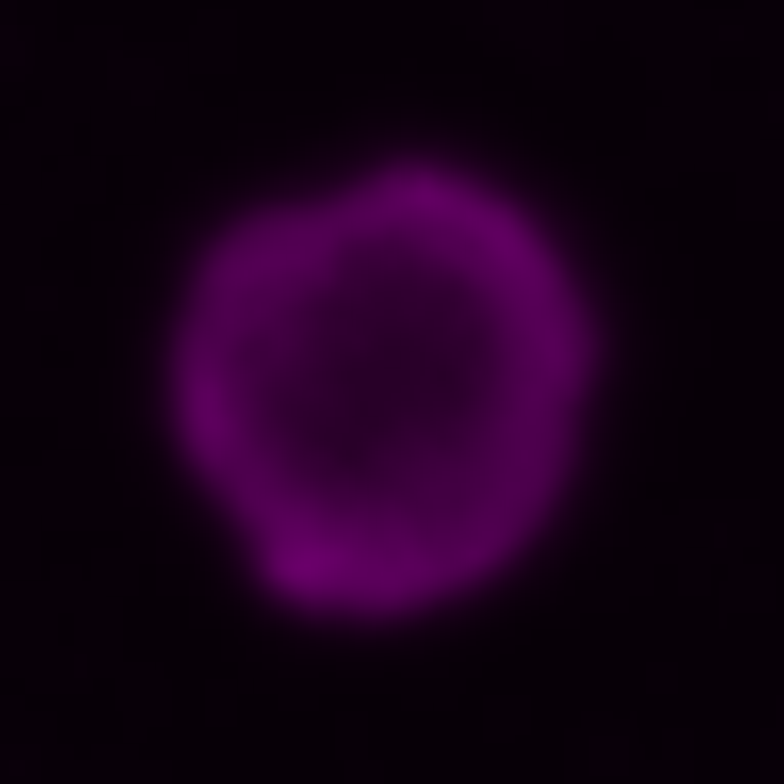

Supplement: Supplementary file 6 — Source data Fig. 4 [file 44319_2025_634_MOESM6_ESM.zip › Figure 4/Fig 4C Anti-H4K16ac/Non-Aged/H4K16ac-Non-aged-010.nd2.tif]

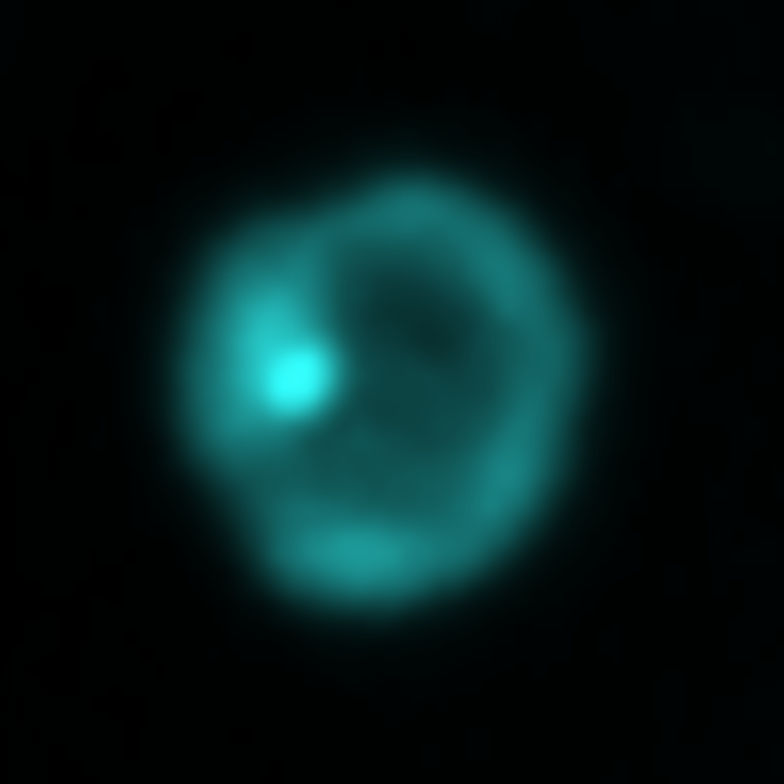

Supplement: Supplementary file 6 — Source data Fig. 4 [file 44319_2025_634_MOESM6_ESM.zip › Figure 4/Fig 4C Anti-H4K16ac/Non-Aged/DNA-Non-aged-010.nd2.tif]

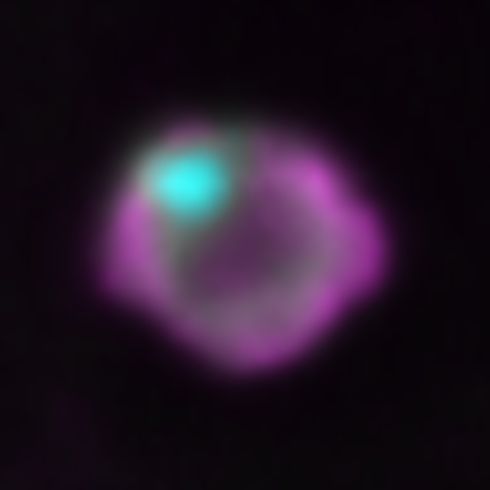

Supplement: Supplementary file 6 — Source data Fig. 4 [file 44319_2025_634_MOESM6_ESM.zip › Figure 4/Fig 4C Anti-H4K16ac/Sirt1 null/Merge-SIRT1 null-006.nd2.tif]

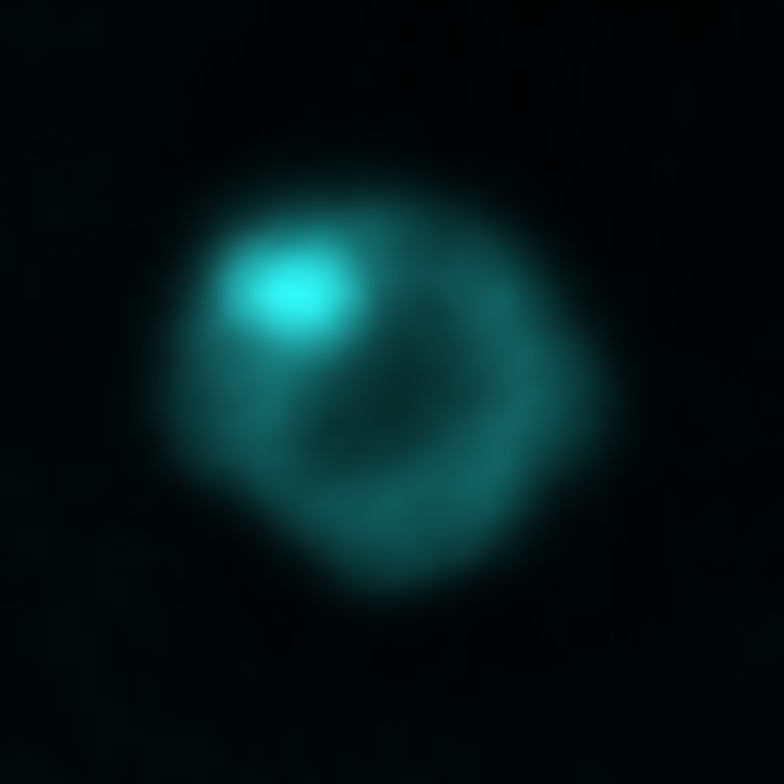

Supplement: Supplementary file 6 — Source data Fig. 4 [file 44319_2025_634_MOESM6_ESM.zip › Figure 4/Fig 4C Anti-H4K16ac/Sirt1 null/DNA-SIRT1 null-006.nd2.tif]

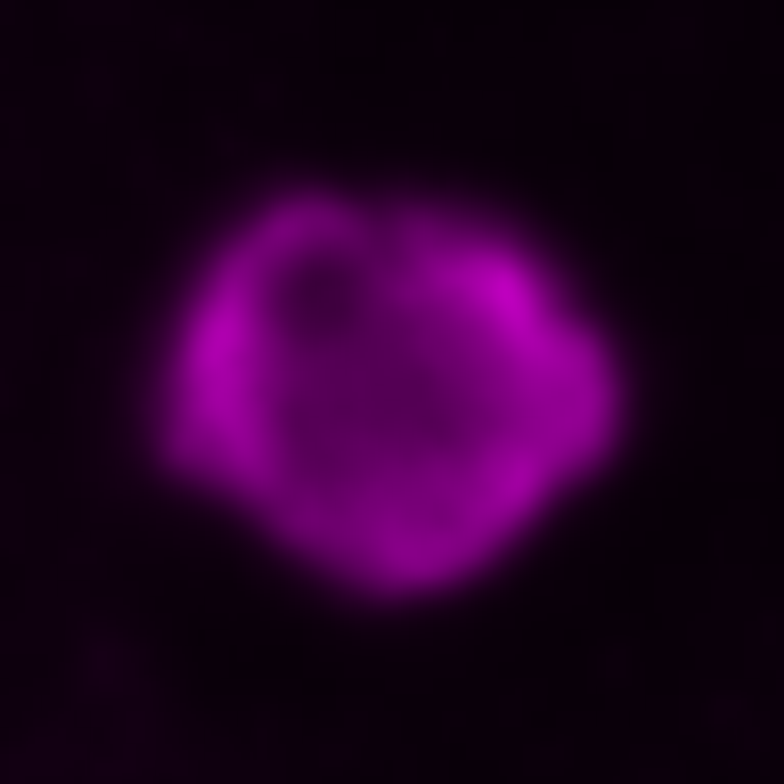

Supplement: Supplementary file 6 — Source data Fig. 4 [file 44319_2025_634_MOESM6_ESM.zip › Figure 4/Fig 4C Anti-H4K16ac/Sirt1 null/H4K16ac-SIRT1 null-006.nd2.tif]

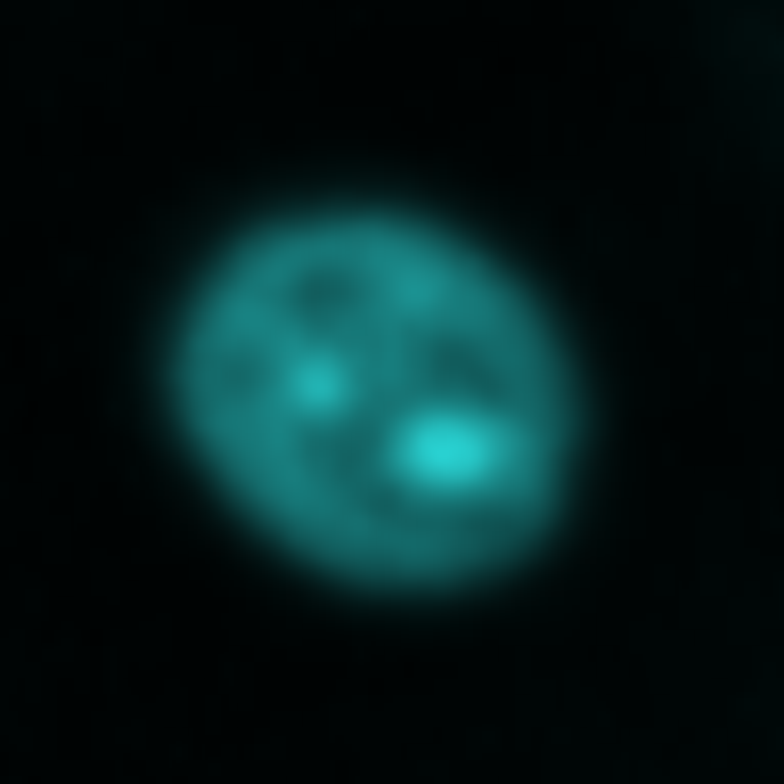

Supplement: Supplementary file 6 — Source data Fig. 4 [file 44319_2025_634_MOESM6_ESM.zip › Figure 4/Fig 4C Anti-H4K16ac/Aged/DNA-Aged-008.nd2.tif]

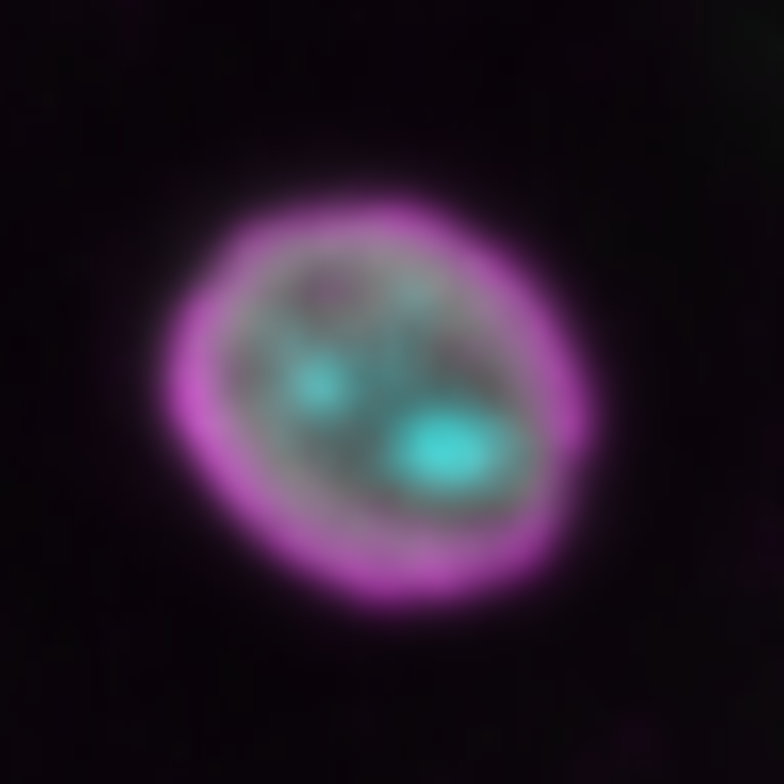

Supplement: Supplementary file 6 — Source data Fig. 4 [file 44319_2025_634_MOESM6_ESM.zip › Figure 4/Fig 4C Anti-H4K16ac/Aged/Merge-Aged-008.nd2.tif]

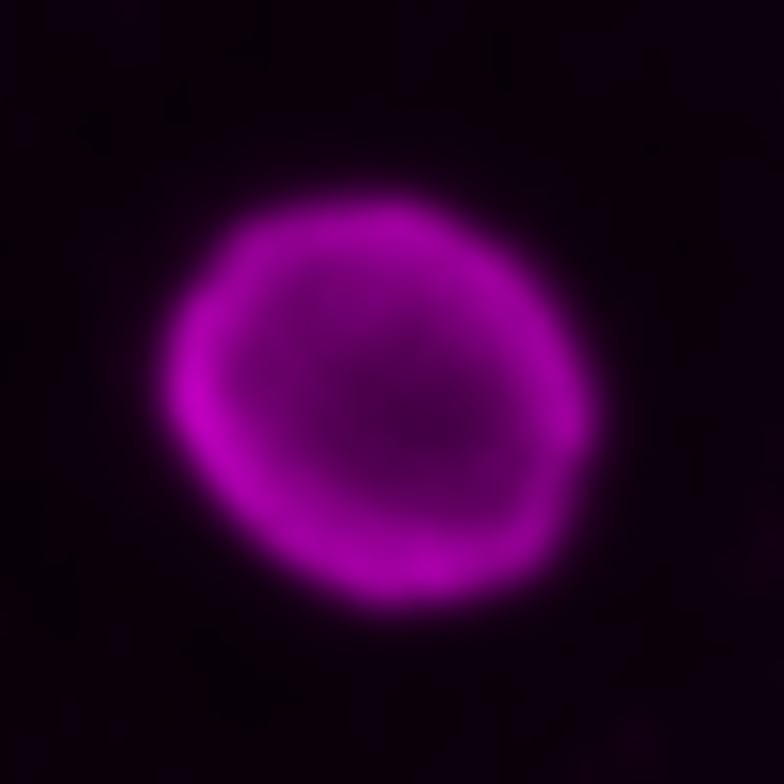

Supplement: Supplementary file 6 — Source data Fig. 4 [file 44319_2025_634_MOESM6_ESM.zip › Figure 4/Fig 4C Anti-H4K16ac/Aged/H4K16ac-Aged-008.nd2.tif]

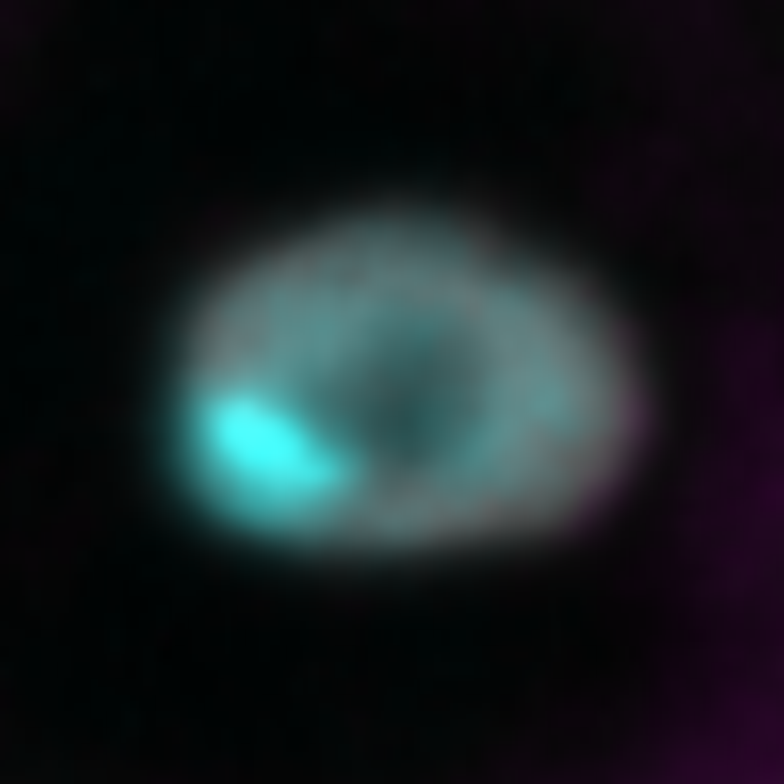

Supplement: Supplementary file 7 — Source data Fig. 5 [file 44319_2025_634_MOESM7_ESM.zip › Figure 5/Fig 5B/SRT1720 Aged/Merge-Aged SRT1720-013.nd2.tif]

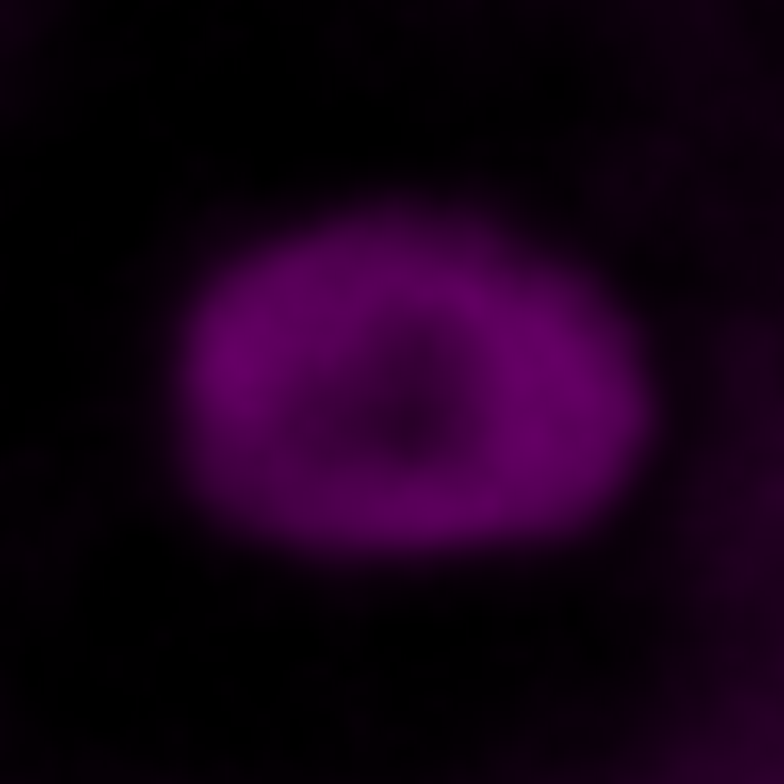

Supplement: Supplementary file 7 — Source data Fig. 5 [file 44319_2025_634_MOESM7_ESM.zip › Figure 5/Fig 5B/SRT1720 Aged/H4K16ac-Aged SRT1720-013.nd2.tif]

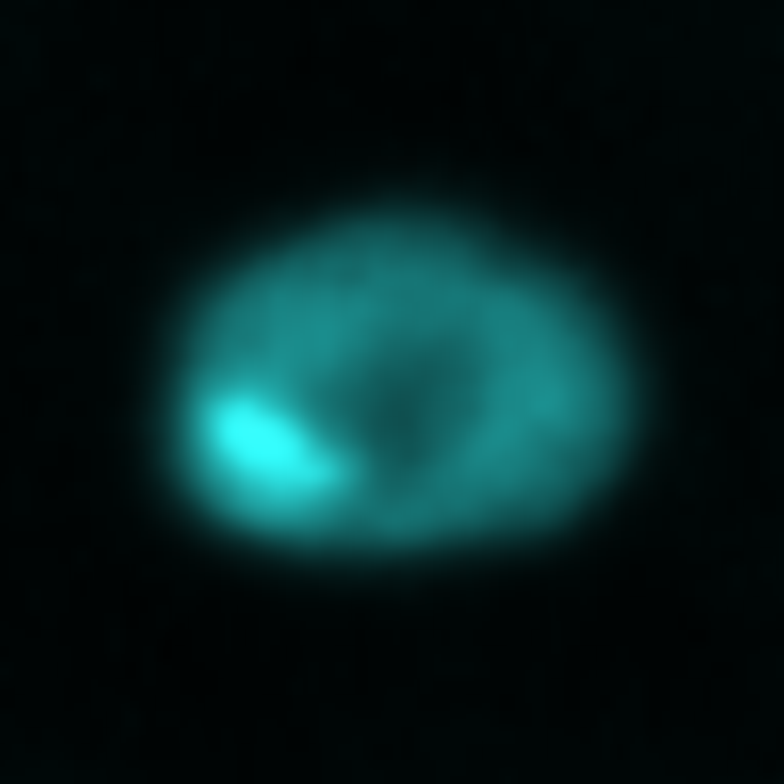

Supplement: Supplementary file 7 — Source data Fig. 5 [file 44319_2025_634_MOESM7_ESM.zip › Figure 5/Fig 5B/SRT1720 Aged/DNA-Aged SRT1720-013.nd2.tif]

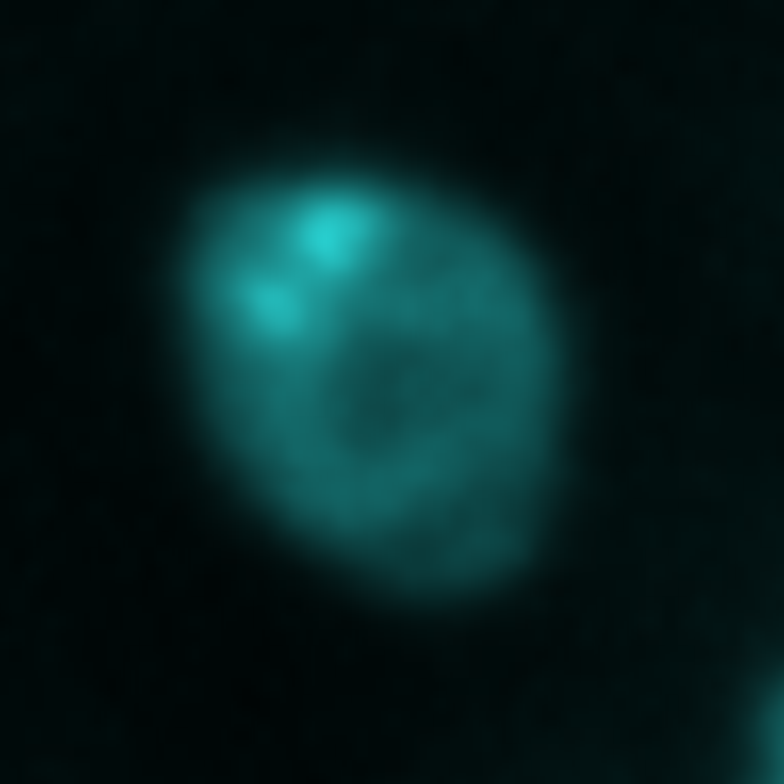

Supplement: Supplementary file 7 — Source data Fig. 5 [file 44319_2025_634_MOESM7_ESM.zip › Figure 5/Fig 5B/DMSO Aged /DNA-Aged-DMSO-9001.nd2-2.tif]

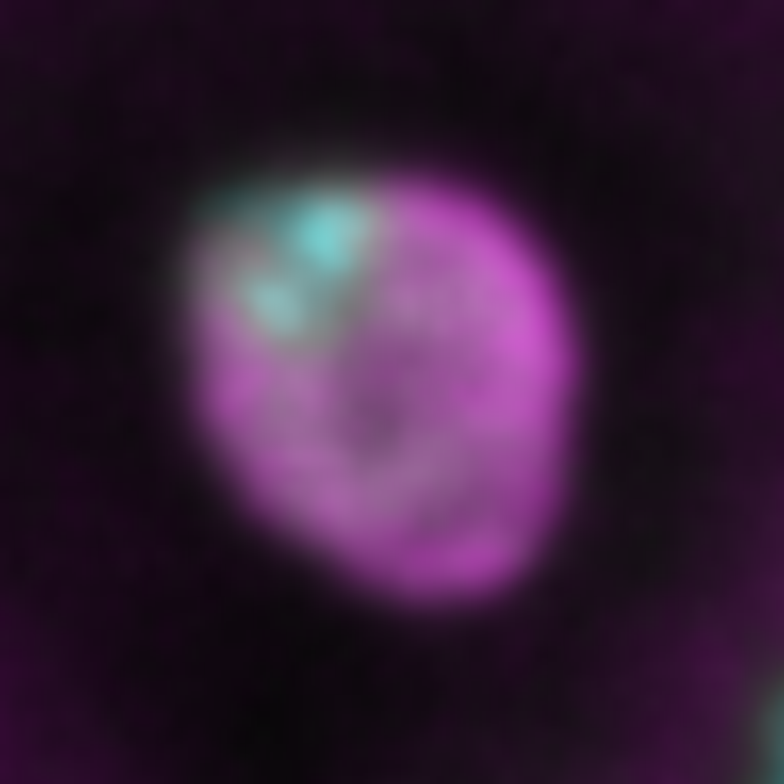

Supplement: Supplementary file 7 — Source data Fig. 5 [file 44319_2025_634_MOESM7_ESM.zip › Figure 5/Fig 5B/DMSO Aged /Merge-Aged-DMSO-9001.nd2-2.tif]

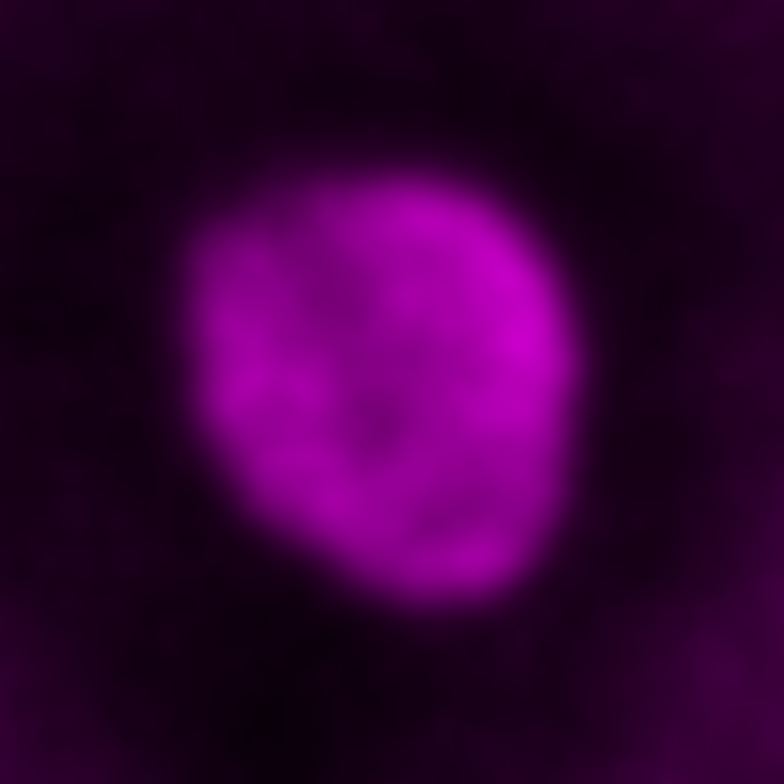

Supplement: Supplementary file 7 — Source data Fig. 5 [file 44319_2025_634_MOESM7_ESM.zip › Figure 5/Fig 5B/DMSO Aged /H4K16c-Aged-DMSO-9001.nd2-2.tif]

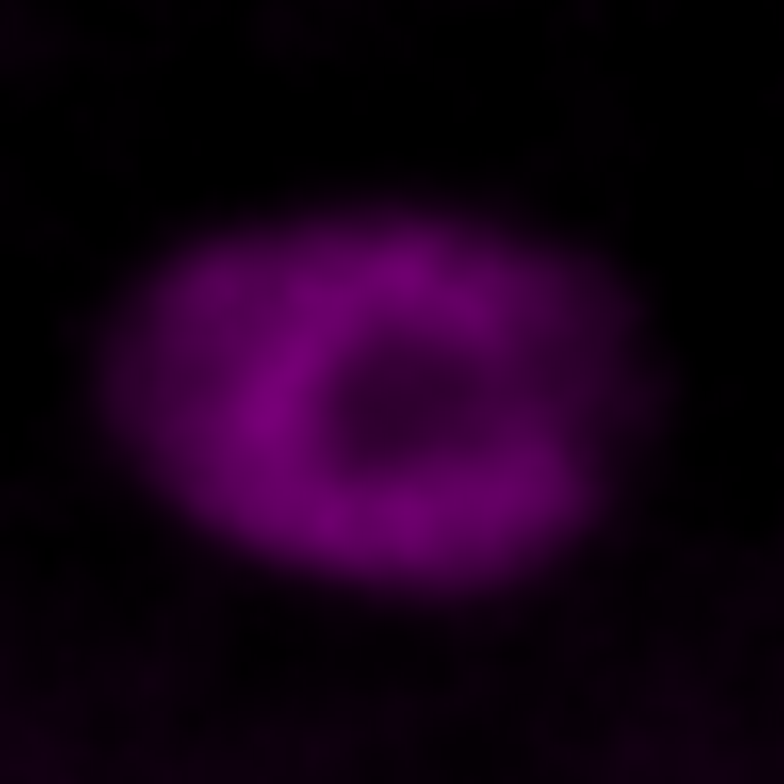

Supplement: Supplementary file 7 — Source data Fig. 5 [file 44319_2025_634_MOESM7_ESM.zip › Figure 5/Fig 5B/SRT1720 Non-aged/H4K16ac-Non-aged SRT1720-012.nd2.tif]

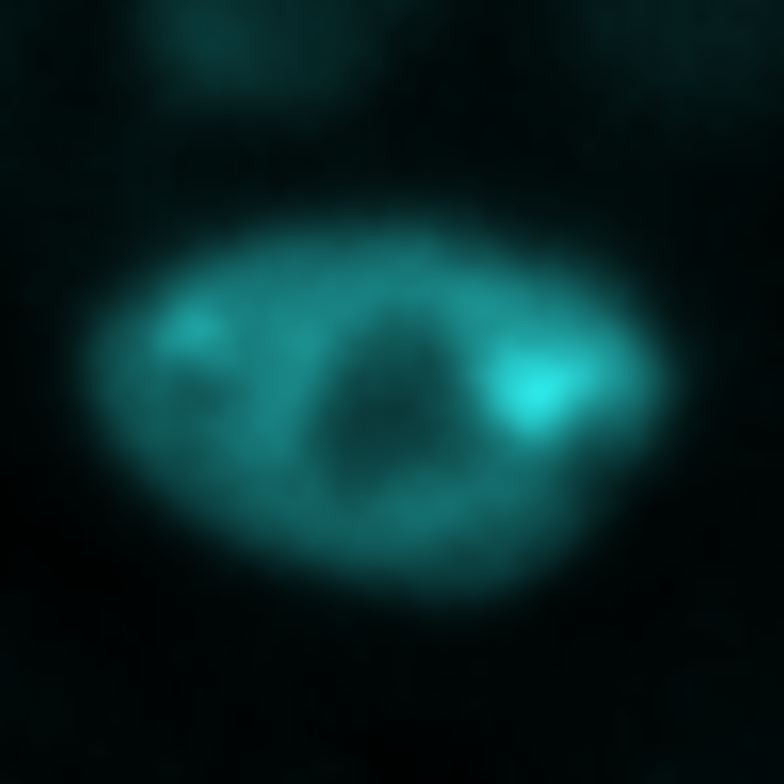

Supplement: Supplementary file 7 — Source data Fig. 5 [file 44319_2025_634_MOESM7_ESM.zip › Figure 5/Fig 5B/SRT1720 Non-aged/DNA-Non-aged SRT1720-012.nd2.tif]

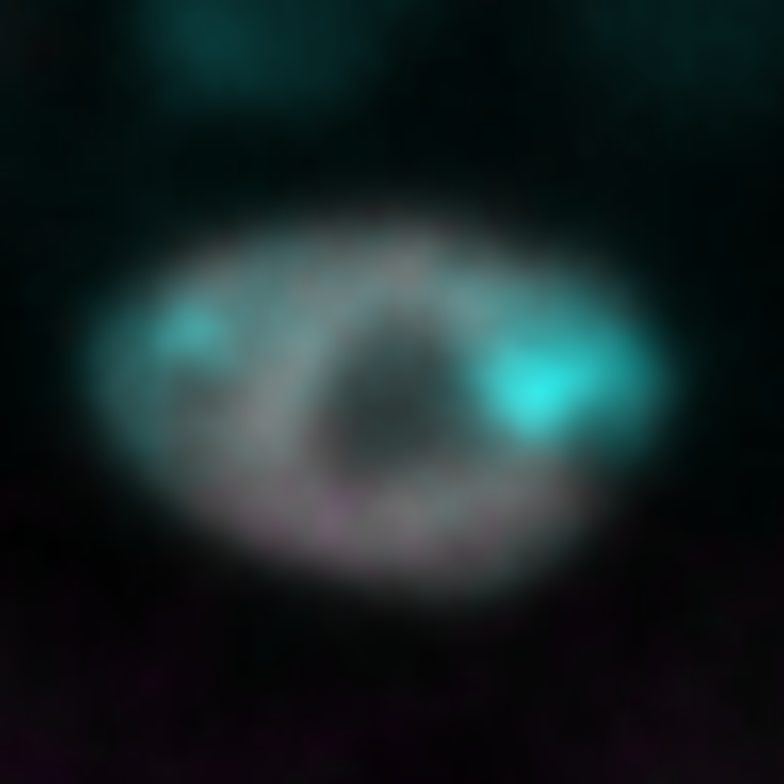

Supplement: Supplementary file 7 — Source data Fig. 5 [file 44319_2025_634_MOESM7_ESM.zip › Figure 5/Fig 5B/SRT1720 Non-aged/Merge-Non-aged SRT1720-012.nd2.tif]

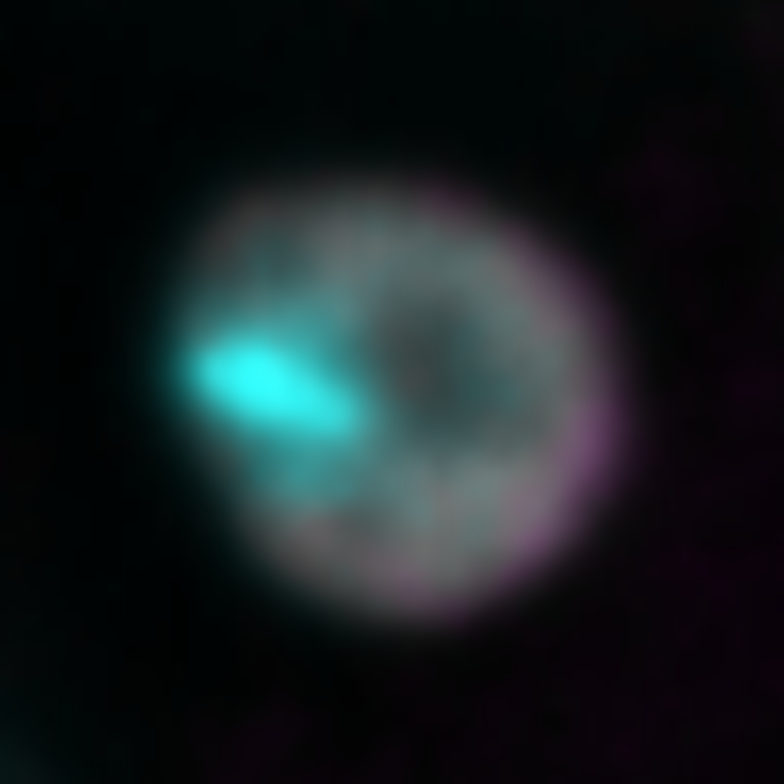

Supplement: Supplementary file 7 — Source data Fig. 5 [file 44319_2025_634_MOESM7_ESM.zip › Figure 5/Fig 5B/DMSO Non-aged/Merge-Non-aged DMSO-018.nd2.tif]

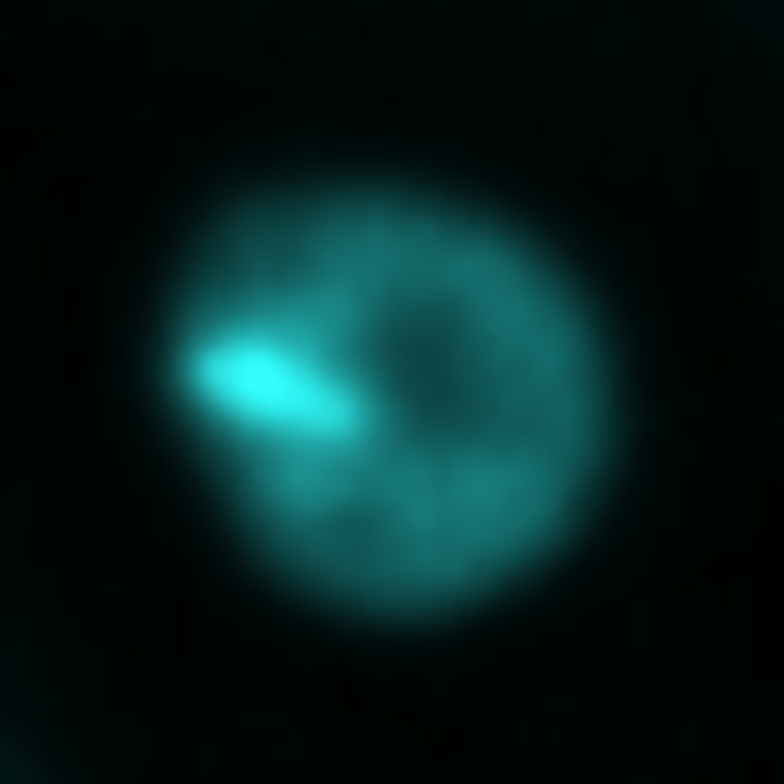

Supplement: Supplementary file 7 — Source data Fig. 5 [file 44319_2025_634_MOESM7_ESM.zip › Figure 5/Fig 5B/DMSO Non-aged/DNA-Non-aged DMSO-018.nd2.tif]

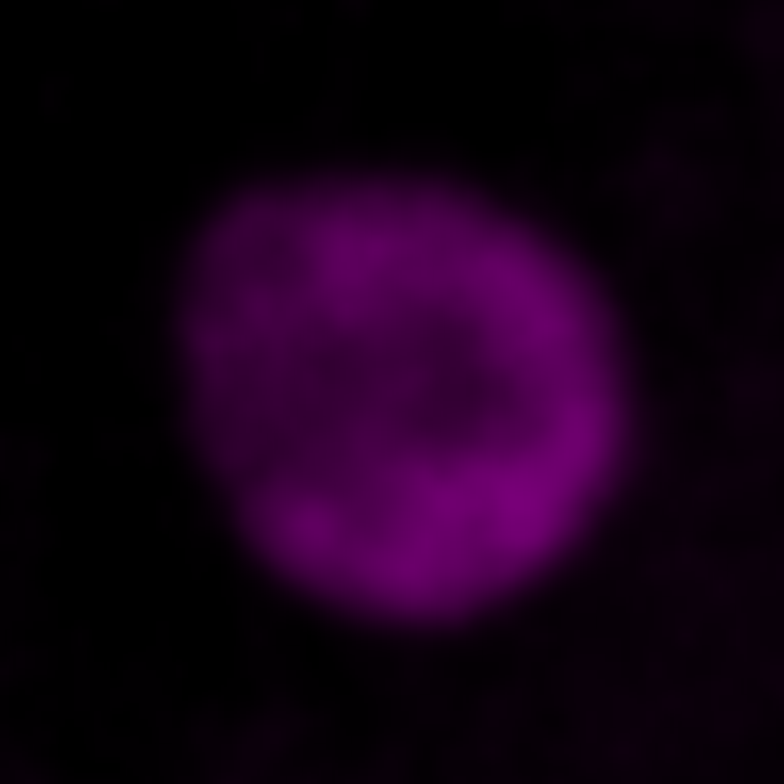

Supplement: Supplementary file 7 — Source data Fig. 5 [file 44319_2025_634_MOESM7_ESM.zip › Figure 5/Fig 5B/DMSO Non-aged/H4K16ac-Non-aged DMSO-018.nd2.tif]

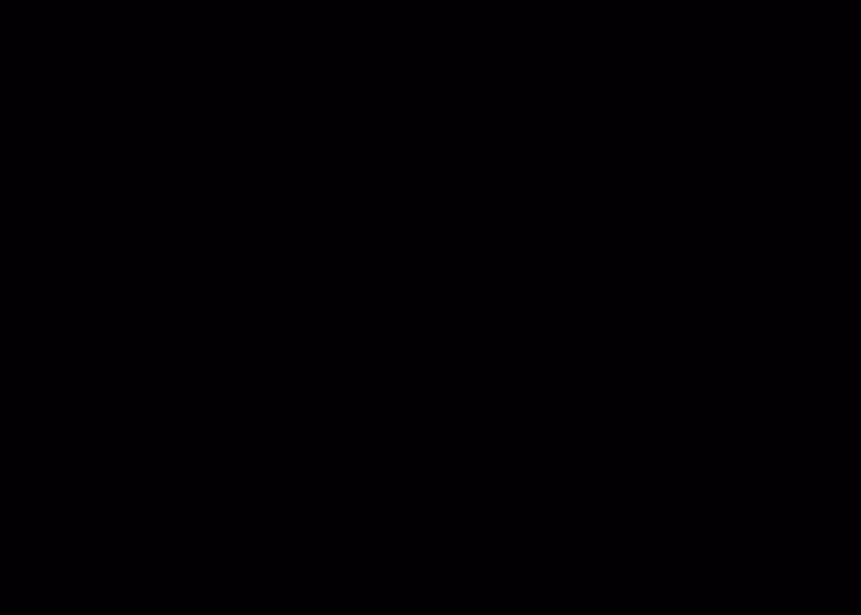

Supplement: Supplementary file 8 — Figure EV1D Source Data [file 44319_2025_634_MOESM8_ESM.zip › Figure EV1D/2. 0-4095 CE Background-006.nd2 (cropped) view as TIFF.tif]

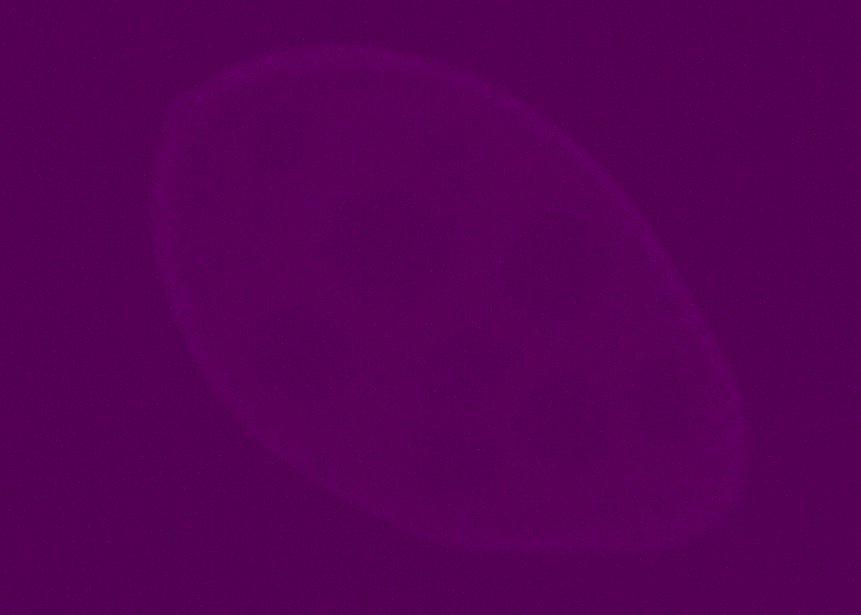

Supplement: Supplementary file 8 — Figure EV1D Source Data [file 44319_2025_634_MOESM8_ESM.zip › Figure EV1D/3. 0-250 CE Background-006.nd2 (cropped) view as TIFF.tif]
