# Supplementary material for: Meiotic cohesion requires Sirt1 and preserving its activity in aging oocytes reduces missegregation
Source: EMBO Rep. 2025 Nov 10;26(24):6121–40. doi: 10.1038/s44319-025-00634-y (PMC12714828; doi:10.1038/s44319-025-00634-y)
Supplement: Supplementary file 8 — Figure EV1D Source Data [file 44319_2025_634_MOESM8_ESM.zip › Figure EV1D/0. Read Me.rtf]

1. Nikon Elements original Z series for EV1D.  All three channels.  Viewable with Image J or FIJI2.  H4K16ac channel only.  Maximum intensity projection.  Contrast enhance 0-4095 (unchanged)3. H4K16ac channel only.  Maximum intensity projection.  Increased contrast enhance to 0-250 shows the egg chamber.  Note that in EV1, the egg chamber has been rotated so that all egg chambers have their posterior on the right.  4. Quantification of H3K16ac (561nm) and Sirt1 (647nm) on oocyte DNA and also in a similar sized volume nearby.  H4K16ac mean signal/voxel is very similar for the DNA volume and the purple square volume. 
