# Supplementary material for: Meiotic cohesion requires Sirt1 and preserving its activity in aging oocytes reduces missegregation
Source: EMBO Rep. 2025 Nov 10;26(24):6121–40. doi: 10.1038/s44319-025-00634-y (PMC12714828; doi:10.1038/s44319-025-00634-y)
Supplement: Supplementary file 9 — Expanded View Figures [file 44319_2025_634_MOESM9_ESM.pdf]

Expanded View Figures

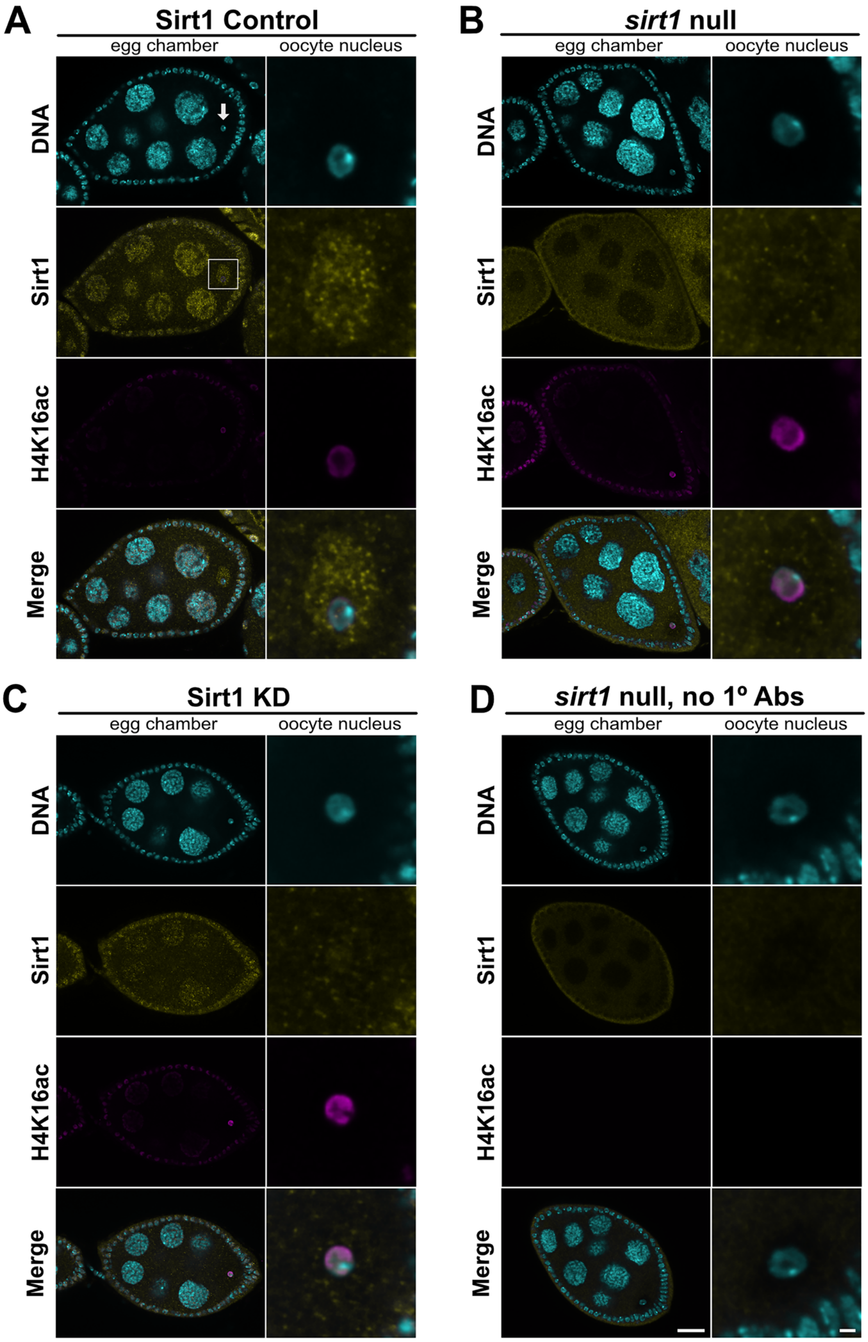

Figure EV1. Sirt1 and H4K16ac immunolocalization in stage 7 egg chambers.

(**A–D**) A single confocal optical section of a stage 7 egg chamber is shown for *Sirt1*$^{SH022-B06}$ control (no driver), KD (matα driver) and *sirt1* null. Anterior on the left. DNA (cyan), Sirt1 (yellow) and H4K16ac (magenta). Scale bars: 20 µm for egg chamber, 2 µm for oocyte nucleus. (**A**) Arrow points to the oocyte DNA and a white square surrounds the oocyte nucleus which is enlarged and shown to the right of each egg chamber image. (**D**) Primary antibodies were omitted when staining *sirt1* null ovaries. Source data are available online for this figure.

 © The Author(s)

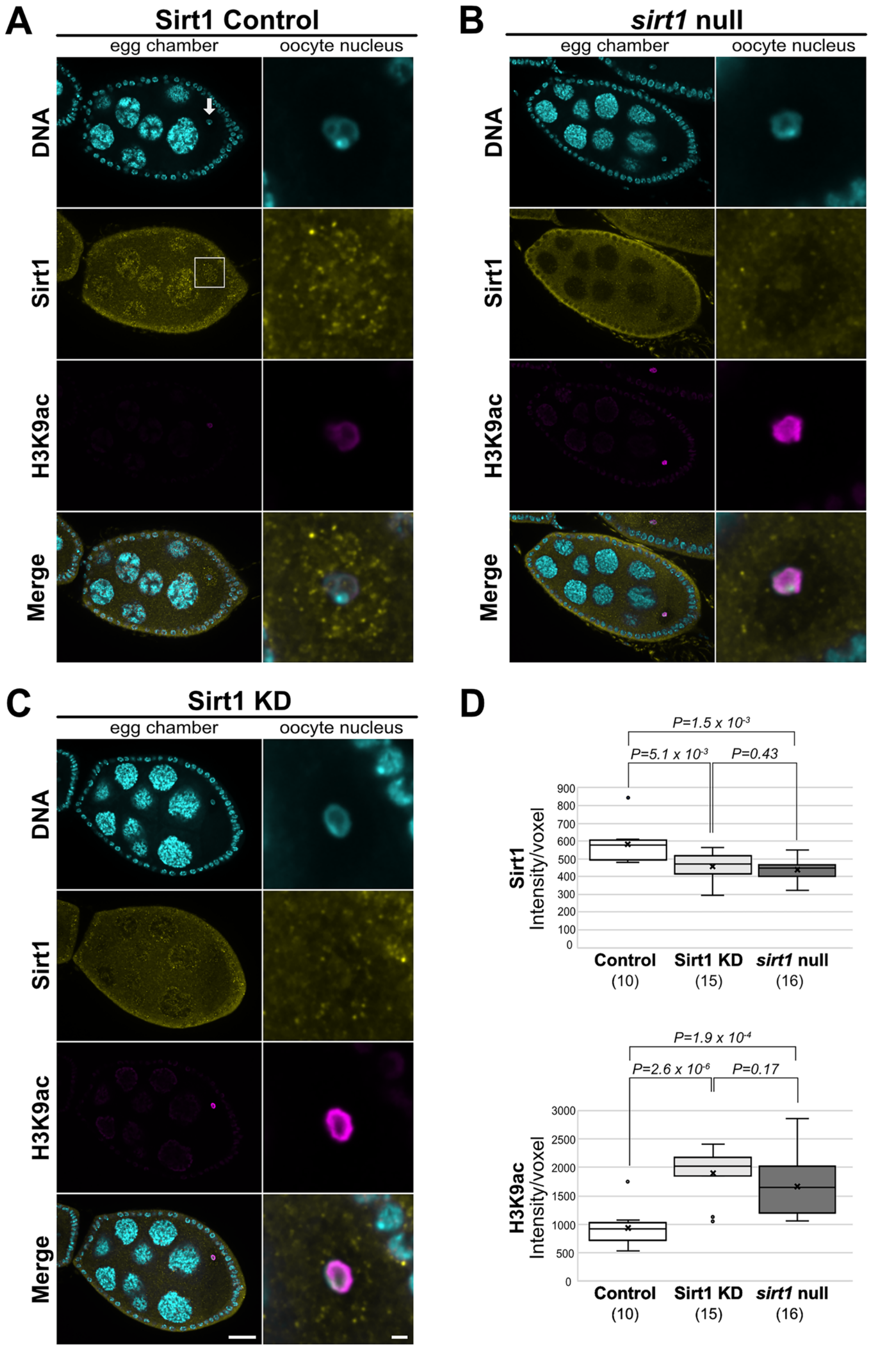

Figure EV2. H3K9 acetylation state in stage 7 oocytes mimics that of H4K16ac.

(**A–C**) A stage 7 egg chamber is shown for $Sirt1^{SH022-B06}$ control (no driver), Sirt1 KD (matα driver), and *sirt1* null. Anterior on the left. DNA (cyan), Sirt1 (yellow), and H4K16ac (magenta). Single confocal optical section. Scale bars: 20 μm for egg chamber, 2 μm for oocyte nucleus. (**A**) An arrow indicates the oocyte DNA, and the oocyte nucleus is surrounded by a white box, which is enlarged and shown next to each egg chamber image. (**D**) Graphs quantify Sirt1 or H3K9ac signal colocalizing with oocyte DNA in the indicated genotypes. Number of oocytes analyzed shown in parentheses. An X marks the average with horizontal lines depicting the median and quartiles. Potential outliers are denoted with a solid black dot. Significance determined with a two-tailed *t*-test.

© The Author(s)

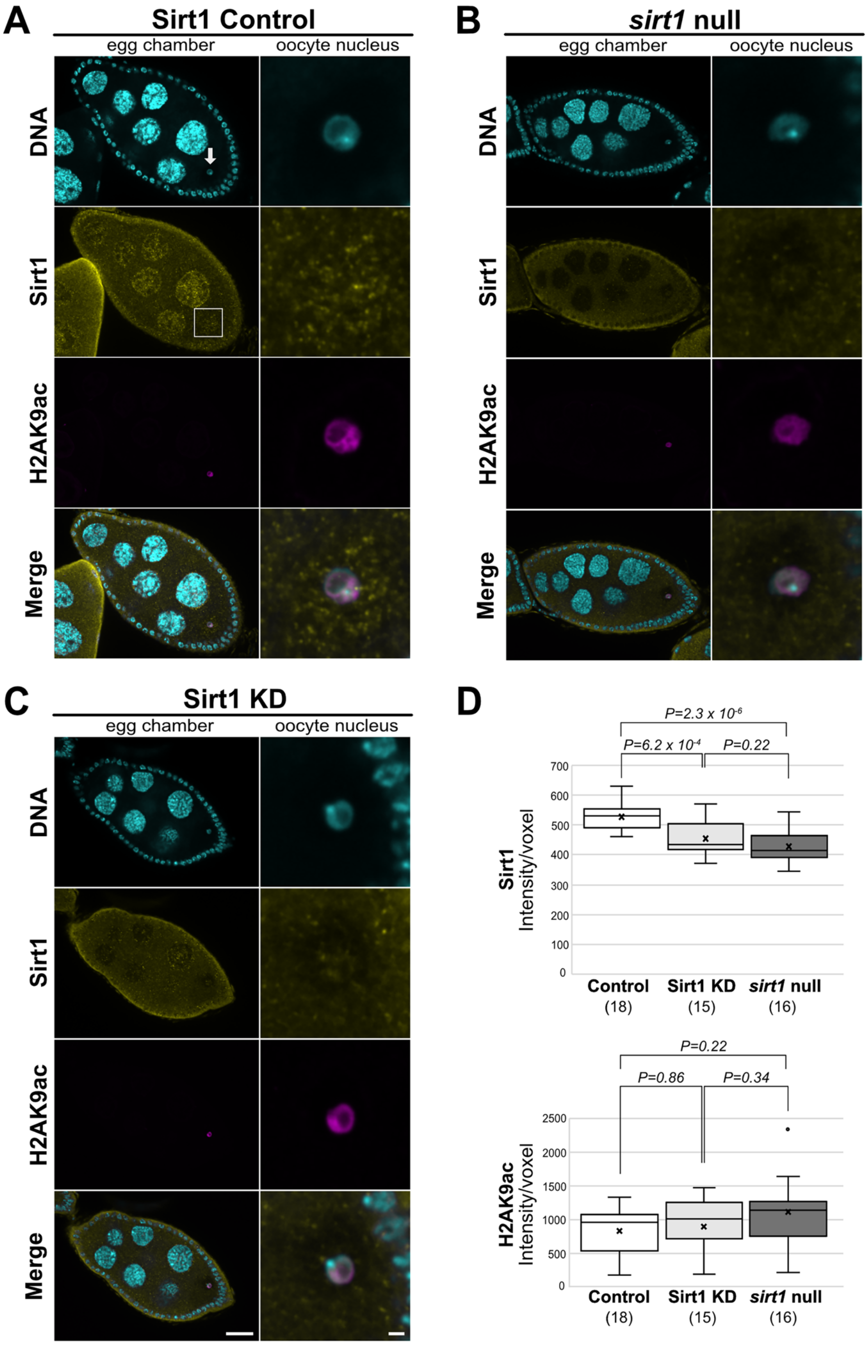

Figure EV3. H2AK9ac on oocyte DNA is not impacted by reduced Sirt1 activity.

(**A–C**) A stage 7 egg chamber is shown for *Sirt1*$^{SH022\text{-}B06}$ control (no driver), Sirt1 KD (matα driver) and *sirt1* null. Anterior on the left. DNA (cyan), Sirt1 (yellow), and H4K16ac (magenta). Single confocal optical section. Scale bars: 20 μm for egg chamber, 2 μm for oocyte nucleus. (**A**) The arrow points to the oocyte DNA and the oocyte nucleus is surrounded by a white box which is enlarged and shown for each egg chamber. (**D**) Graphs quantify Sirt1 or H2AK9ac signal colocalizing with oocyte DNA in the indicated genotypes. Number of oocytes analyzed shown in parentheses. The average is denoted with an X, and horizontal lines mark the median and quartiles. Potential outliers are denoted with a solid black dot. Significance determined with a two-tailed *t*-test.

 © The Author(s)

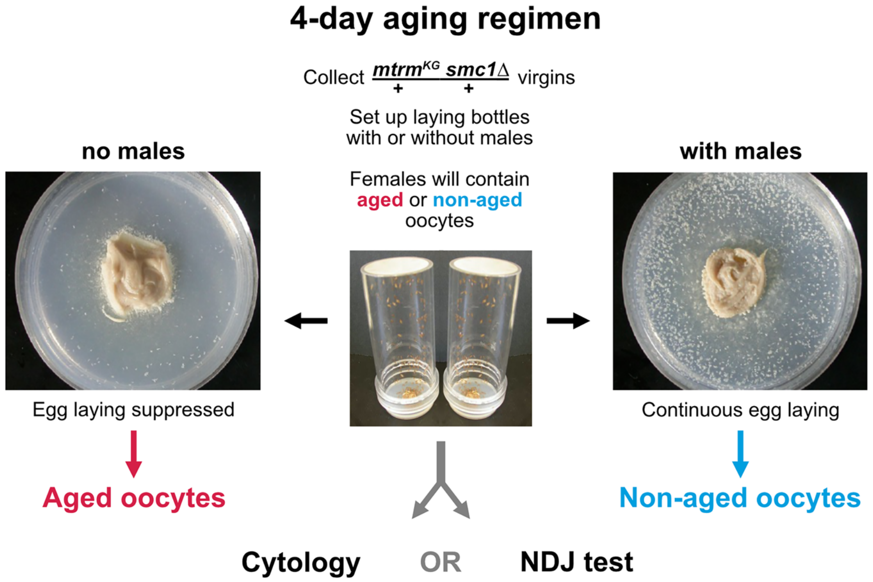

Figure EV4. Experimental procedure to generate and compare aged and non-aged oocytes.

mtrm^{KG} *smc1Δ*/+ virgins are placed in plastic laying bottles with a glucose/agar plate and yeast paste and *X^Y, B* males are omitted (left, aged) or added (right, non-aged). Left: In the absence of mating, egg laying is suppressed and most oogenesis stages halt progression. On the agar plate shown, very few unfertilized eggs (white spots on the agar surface) have been laid during the 24-h interval. When oogenesis halts, oocytes arrest and age at a particular stage. *Drosophila* oocytes are vulnerable to aging-induced segregation errors when they arrest and age in diplotene. Right: Oogenesis is stimulated in females that have mated, and many fertilized eggs are laid within a 24-h period. Because oogenesis is continuous in these females, they produce non-aged oocytes.

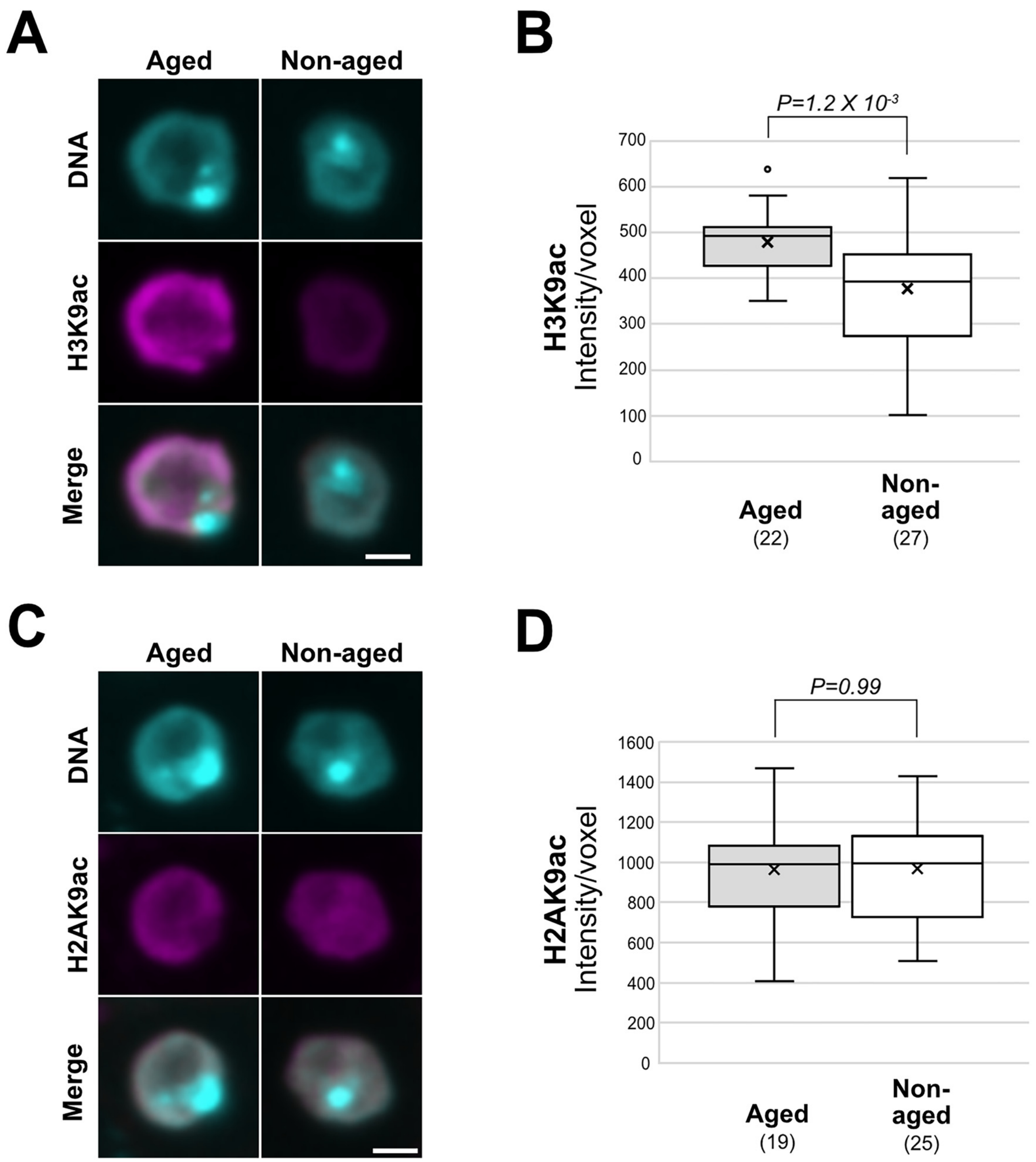

Figure EV5. Acetylation of H3K9 but not H2AK9 increases on oocyte DNA during aging.

(**A**, **C**) H3K9ac or H2AK9ac immunostaining (magenta) on the DNA (cyan) of stage 7 aged and non-aged oocytes. All images are maximum intensity projections of confocal Z series. Scale bar, 2 µm. (**B**) Like the Sirt1 substrate H4K16ac, aging causes a significant increase in H3K9 acetylation on oocyte DNA, consistent with loss of Sirt1 deacetylase activity during aging. (**D**) Aging does not alter the H2AK9ac signal intensity on oocyte DNA. The number of oocytes scored in (**B**, **D**) is shown in parentheses. *P* values were determined using a two-tailed unpaired *t*-test. (**B**, **D**) An X indicates the average, with horizontal lines denoting the median and quartiles. Potential outliers are denoted with a solid black dot.

 © The Author(s)
